# Cryo-EM structures of DNA-free and DNA-bound BsaXI: architecture of a Type IIB restriction–modification enzyme

**DOI:** 10.1093/nar/gkaf291

**Published:** 2025-04-15

**Authors:** Betty W Shen, Dan Heiter, Weiwei Yang, Shuang-yong Xu, Barry L Stoddard

**Affiliations:** Division of Basic Sciences, Fred Hutchinson Cancer Research, 1100 Fairview Ave. N. Seattle, WA 98109, United States; New England Biolabs, 240 County Road Ipswich, MA 01938, United States; New England Biolabs, 240 County Road Ipswich, MA 01938, United States; New England Biolabs, 240 County Road Ipswich, MA 01938, United States; Division of Basic Sciences, Fred Hutchinson Cancer Research, 1100 Fairview Ave. N. Seattle, WA 98109, United States

## Abstract

We have determined multiple cryogenic electron microscopy (cryo-EM) structures of the Type IIB restriction–modification enzyme BsaXI. Such enzymes cleave DNA on both sides of their recognition sequence and share features of Types I, II, and III restriction systems. BsaXI forms a heterotrimeric (RM)_2_S assemblage in the presence and absence of bound DNA. Two unique structural motifs—a multi-helical “knob” and a long antiparallel double-helical “paddle”—are involved in DNA binding and cleavage. Binding of the DNA target triggers a large conformational change from an ‘open’ to ‘closed’ configuration, resulting in a mixture of two different conformations with respect to the positioning of the S subunit and its target recognition domains on the enzyme’s bipartite DNA target site. Structure-guided mutagenesis studies implicated two clusters of residues in the RM subunit as being critical for DNA cleavage, both are located proximal to a DNA cleavage site. One corresponds to a canonical PD-(D/E)xK endonuclease site in the N-terminal endonuclease domain, while the other corresponds to residues clustered within the paddle motif (near to the C-terminal end of the RM subunit). This analysis facilitates a comparison of three potential mechanisms by which such enzymes cleave DNA on each side of the bound target.

## Graphical Abstract

**Graphical Abstract:**
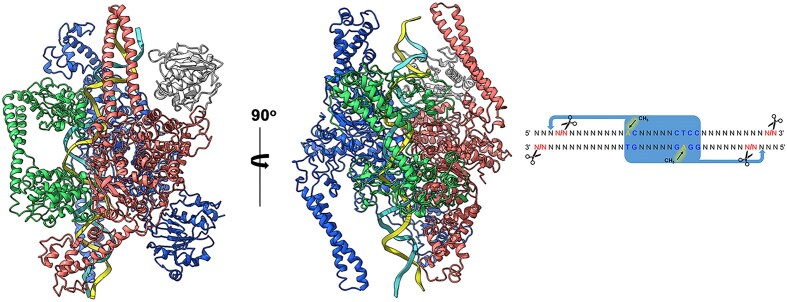

## Introduction

Restriction–modification (RM) systems are one of a bacterium’s first lines of defense against phage infections (alongside adaptive CRISPR systems). They act by cleaving invading phage DNA within or near precisely defined, short target sites via the action of a restriction endonuclease (REase) subunit or domain, while simultaneously protecting host DNA against cleavage at the same sites via the corresponding action of a cognate methyltransferase activity (MTase) [1–3]. Most bacterial genomes encode multiple RM systems, that are loosely classified into four different major groups (Types I, II, III, and IV) based on their structural organization, their modes of action, and their cofactor requirements [4–6]. While Types I, II, and III systems act upon unmodified target sites, Type IV enzymes cleave modified target sites that are generated within phage genomes as a resistance mechanism against RM action and restriction [7].

Type I and Type III systems [8, 9] are multi-functional ATP-dependent enzymes, usually comprised of multiple subunits, that couple site-specific DNA recognition, DNA cleavage, and DNA methylation activities to the action of a translocase domain that mobilizes the bound enzyme complex along the DNA duplex. They cleave DNA at positions located either at precisely defined phosphate groups near their target sites (Type III) or at variable, more distant positions relative to their target sites (Type I). DNA cleavage activity is usually triggered by collision and interaction between two enzyme complexes that were (or still are) bound to separate individual targets [10].

Type I, III, and IV systems represent well over half of RM systems found in nature [2], and they generally must engage multiple unmodified target sites before cleaving DNA. The most common of these enzyme systems (Type I) are usually encoded by three consecutive genes (*Hsd*R, *Hsd*M and *Hsd*S) that respectively encode subunits that correspond to endonuclease (‘REase’ or ‘R’), methyltransferase (‘MTase’ or ‘M’) and site-specific DNA recognition and binding (‘S’) functions [8]. The R subunit typically also contains the translocase domain, and the protein subunits usually form a multifunctional R_2_M_2_S complex. While most characterized Type I enzymes follow this pattern, many exceptions exist that combine these functional domains in different combinations across individual protein chains, including single chain ‘RMS’ subunits.

In contrast, Type II RM systems [11] do not rely upon ATP-dependent translocase domains, and have provided investigators with the most extensively studied and broadly utilized restriction endonucleases. They identify and bind to specific target sequences via target search mechanisms that rely on short-lived encounters with DNA that are generated via random diffusion and hopping between chromosomal regions [12], eventually leading to a target binding and cleavage either within or near their recognition sites. The structure and organization of Type II RM systems are also extremely diverse, ranging from the stand-alone enzymes that act at either palindromic or asymmetric targets (such as EcoRI or FokI, respectively) to longer, chimeric polypeptides that organize several independently folded, functional domains into multifunctional RM enzyme complexes that contain both endonuclease and methylase activities (such as BpuSI, MmeI, and DrdV) [11].

One quite unique class of Type II RM enzymes—“Type IIB”, exemplified by the archetypical enzyme BcgI [13, 14]—has been found to display a combination of properties associated with Type I, II, and III systems [15, 16]. Much like Type I systems, most of these enzymes are comprised of multiple protein chains that encompass R, M, and S functions, and recognize bipartite targets with two recognition sequences (each 2–4 base pairs in length) separated by 5–7 variable intervening bases. Like Type III enzymes, they generate double-strand breaks at fixed distances on both sides of the target, leading to the excision of a short fragment, from the surrounding DNA, along with the generation of incompatible DNA overhangs. The two recognition sequences within the target are sometimes related by symmetry, but more often are asymmetric and often of different lengths. Finally, like all Type II systems, Type IIB enzymes do not possess or rely upon an ATP-dependent translocase for locomotion on DNA, and only require S-adenosyl methionine (SAM) and divalent metal ion (i.e. Mg^2+^) for DNA methylation and cleavage, respectively.

The type IIB enzyme R.M.BsaXI (“BsaXI” hereafter) is encoded by two reading frames within the *Bacillus stearothermophilus genome*, that jointly encode a 916-residue RM fusion subunit and a 476-residue S subunit [17]. It recognizes and cleaves around a bipartite target site [denoted by convention as (9/12) ACNNNNNCTCC (10/7); further illustrated below] consisting of two recognition sites (5′-AC-3′ and 5′-CTCC-3′) separated by five variable intervening bases. Cleavage of the top and bottom strands occurs 9 and 12 bases, respectively, upstream of the 5′ end of the target site (generating a 3-base, 3′ overhang on the lower strand) and 10 and 7 bases, respectively, downstream of the 3′ end of the target site (also generating a 3 base, 3′ overhang, on the top strand):

- 5′ N N N N**/**N N N N N N N N N **A C** N N N N N **C T C C** N N N N N N N N N N**/**N 3′
- 3′ N**/**N N N N N N N N N N N N **T G** N N N N N **G A G G** N N N N N N N**/**N N N N 5′

These two cleavage events thereby generate a 27 base pair excised DNA fragment with a 3 nucleotide (nt) 3′ overhang on each end. A wealth of previously described biochemical and structural studies on related enzymes, that each depend on an S subunit for DNA recognition, suggests that the two halves of the bipartite recognition site (5′-AC-N_5_-CTCC-3′) are engaged by the two target recognition domains (TRDs) located at opposite ends of the S subunit, whereas the RM fusion subunits are responsible for DNA cleavage and/or methylation. However, the stoichiometry, subunit organization and architecture, and mechanism resulting in four strand scission events (i.e. two DNA cleavage events) by the DNA-bound active enzyme complex and the mechanism of action are not well-established.

In this study, we describe a series of cryogenic electron microscopy ('cryo-EM') structures of BsaXI, both in its DNA-free form and in two closely related DNA-bound forms. We identify two adenine bases (one on each strand, respectively located in the 5′ and 3′ half-sites) that are each flipped out and targeted for methylation by the enzyme, demonstrate that hemi-methylation at either position is sufficient to prevent cleavage on either side of the target, map the location of residues near the cleavage sites that are critical for DNA cleavage, and finally discuss possible mechanisms by which BsaXI carries out DNA cleavage and target excision.

## Materials and methods

### Subcloning, expression, and purification

Two different strategies were used to produce BsaXI for structural studies and for biochemical activity assays of a panel of point mutants, respectively, as described in separate methods sections below. Competent cells of the cloning, expression and T7 expression strain C2566 (T7 Express), pUC19 plasmid, phage λ DNA, restriction enzymes, Phusion DNA polymerase, T4 polynucleotide kinase, Quick T4 DNA ligase, plasmid mini-prep kit, and DNA clean-up kit were provided by New England Biolabs (NEB). Deoxyoligonucleotides (oligos) and mutagenic primers for site-directed mutagenesis (Supplementary Table S1) were purchased from IDT. T7 vector pET21b for His-tagged BsaXI S subunit expression was purchased from Novagen.

#### Generation of reconstituted BsaXI for structural studies

For structural studies, each subunit was independently expressed as an N-terminal fusion to a chitin-binding domain and a self-splicing intein and then purified via liberation of the enzyme subunit after an initial affinity capture step, followed by multiple additional chromatographic steps. The cloning, over expression and purification of wild type BsaXI was carried out as previously described [17]. C2566 [pTYB1-*bsaXIRM*] and C2566 [pTYB1-*bsaXIS*] cells were grown in 1 l culture flasks containing Luria-Bertani ('LB') media augmented with 100 mg/l ampicillin and grown at 30°C until OD600 reached 1.3. After cooling to room temperature over 30 min, protein expression was induced via the addition of isopropyl β-D-thiogalactopyranoside ('IPTG') to a final concentration of 0.4 mM and the culture was shaken overnight at 18°C. Cells were harvested by centrifugation and stored at −80°C.

Frozen cell pellets from 3 l of BsaXI RM culture and 6 l of BsaXI S culture were all thawed (preliminary experiment indicated that the expression level of RM subunit was significantly higher than the S subunit), resuspended in chitin column buffer [500 mM NaCl, 20 mM Tri–HCl, pH 8.5, 1 mM ethylenediaminetetraacetic acid (EDTA), and 0.1% Triton X-100], lysed through two passes at 30 000 psi in a Dihydromatics^®^ HL60 cell disruptor, and centrifuged to remove cell debris. Crude supernatants were loaded through two separate 40 ml chitin resin (NEB S6651) columns by gravity, washed with 300 ml of chitin column buffer, and then cleaved over 3 days with 50 mM dithiothreitol ('DTT') chitin cleavage/elution buffer.

All chitin eluates were pooled together in 450 ml, diluted to 300 mM NaCl with addition of 300 ml zero salt column buffer and flowed through 20 ml diethylaminoethyl ('DEAE') resin to remove nucleic acids. DEAE flow-through and wash were again diluted to 150 mM NaCl with zero salt column buffer, applied to a Heparin TSK column (11 ml) and eluted with increasing NaCl gradient. Pooled active fractions were dialyzed overnight against 100 mM NaCl, 20 mM Tris–HCl, pH 8, 1 mM DTT, 0.1 mM EDTA, and 5% glycerol, then passed through a Source 15S column (21 ml), which was applied to 22 ml Source 15Q and eluted with increasing NaCl gradient (0.1–1 M). Pooled active fractions were dialyzed overnight against NEB Diluent B and applied to a Superdex75 SEC column (1787 ml), leading to an elution profile corresponding to a heterotrimeric complex containing two RM subunits and one S subunit [(RM)_2_S]. Pooled active fractions were concentrated by a second run over Heparin TSK, giving a final yield of ∼10.0 mg pure BsaXI REase.

The BsaXI/DNA complexes were prepared both in the [1] presence or [2] absence of the SAM analogue sinefungin, but only the latter preparation was used for subsequent structural studies. The structure of the DNA-free BsaXI clearly contained the cofactor SAM, which suggests that the cofactor copurified with the enzyme as a tightly bound complex and was carried over to the subsequent DNA-bound complexes.

#### Mutagenesis and generation of reconstituted BsaXI mutants for activity studies

A *bsaXIS* polymerase chain reaction (PCR) fragment (comprising the reading frame encoding the S subunit) was amplified and inserted into the NdeI and XhoI sites of pET21b to obtain C-terminal 6xHis tagged protein (6xHis-S subunit). Due to the large size of pTYB1-*bsaXIRM*, a smaller plasmid carrying the RM gene was constructed for the ease of PCR mutagenesis. A *bsaXIRM* PCR fragment (comprising the reading frame encoding the RM subunit) was amplified and inserted into the HindIII and BamHI sites of pUC19 to generate an N-terminal 8xHis-tagged RM subunit. The S and RM subunits were individually purified via Ni-agarose (NEB) affinity chromatography and then reconstituted to assay for endonuclease activity in 1× CutSmart buffer at 37°C for 1 h. (1× CutSmart buffer composition: 50 mM potassium acetate, 20 mM Tris-acetate, 10 mM magnesium acetate, 100 μg/ml BSA. pH 7.9 at 25°C.)

### Sample and grid preparation for electron microscopy

Aliquots of purified BsaXI RM/S complex from NEB in storage buffer containing 50% glycerol and 25 mM DTT were combined and exchanged into Buffer A (20 mM Tris–HCl, pH 8.0, 150 mM NaCl, 1 mM EDTA, 1 mM DTT) during fractionation over a SuperDex600 SEC column to eliminate aggregates and uncomplexed RM and S subunits. The center fractions of the major peak were pooled, concentrated down to ∼10 mg/ml, and stored at −80°C in 50 μl aliquots (Supplementary Fig. S1A, left panel).

To generate a DNA-bound complex of BsaXI, 163 μl of Buffer B (20 HEPES, pH 7.5 containing 150 mM NaCl, 2 mM CaCl_2_, and 1 mM DTT) and 13 μl of sinefungin at 26.6 mM (final concentration 1 mM) or d/dH_2_O was added to 300 μl of DNA-free BsaXI at ∼9 mg/ml (OD260/OD280 = 0.57). The mixture was divided into two 238 μl tubes. To one of the sample was added 12 μl of a duplex containing the enzyme’s target site (at 500 micromolar concentration) with the sequence:

- 5′-AATAAGCTGAATATTGTCGGA**AC**CAAGT**CTCC**ATATGGAATTAATAAGCTAG-3′

and its complement

- 3′-TTATTCGACTTATAACAGCCT**TG**GTTCA**GAGG**TATACCTTAATTATTCGATC-5′

where the underlined base-pairs indicate the bipartite target site.

As a control for the final size exclusion run on the protein–DNA complex, 12 μl of TE were added (in place of the DNA duplex) to the other sample. Both samples were incubated at room temperature for 2 h prior to fractionation on a Bio-Rad Enrico650 SEC size-exclusion (Supplementary Fig. S1A, right panel). The center fractions of the peak corresponding to BsaXI/DNA complex (Peak I) were pooled and concentrated to ∼1 mg/ml (final OD^280^ = 1.56 and OD^260^/OD^280^ = 1.16), flash-frozen in 25 μl aliquots, and stored at −80°.

### Initial evaluation via negative stain Transmission Electron Microscopy (TEM)

Negative-stain grids were prepared by the application of 4 μl of size exclusion chromatography ('SEC') purified samples to a glow discharged uniform carbon film coated grid. The particles were allowed to adsorb to the film for 60 s. Excess solution was removed by briefly touching the edge of a filter paper. The grid was quickly washed three times with 20 μl drops of water and once with a 20 μl drop of 0.5% uranyl formate (UF) followed by staining for 20 s in a 40 μl drop of UF. The grids were air-dried for at least 2 h prior to inspection on an in-house JEOL JM140 microscope (operating at 120 kV) equipped with a Gatan Rio 4kx4k CMOS detector. Both DNA-free and DNA-bound particles distributed homogeneously in random orientations over the surface of the carbon film in negative stained preparations. The DNA-free particles are more elongated and flexible (Supplementary Fig. S1A, left, inset) than are the more compact DNA-bound particles (Supplementary Fig. S1A, right, inset). Preliminary assessment of the feasibility of the project was conducted by the reconstruction/refinements of a ∼15 Å molecular envelop for the DNA-free BsaXI from a small ns-TEM dataset of 250 micrographs using 12 800 selected particles after two rounds of 2D classification/selection jobs, followed by 3D *ab initio* reconstruction of a single model, and homogenous plus nonuniform refinements (Supplementary Fig. S1B).

### Cryogenic electron microscopy

Screening for vitrification conditions and initial screening using a Glacios electron microscope (operating at 200 kV). A full data set for DNA-free samples was collected on an FEI Krios electron microscope (operating at 300 kV) at the Pacific North cryo-EM Center (PNCC). Three data sets for the DNA-bound samples were collected, two on the in-house 200 kV Glacios microscope and one on a Krios at PNCC. The DNA-bound datasets were combined after scaling together using the script boxscaler.py [18].

All data preprocessing, which included motion correction, ctf estimation, and exposure curation, as well as 2D particle classification/selection, 3D-model reconstruction/refinement, and post refinement were performed using the software package cryoSPARC2 [19]. For each movie stack, the frames were aligned for beam-induced motion correction using Patch-motion-correction. Patch-CTF was used to determine the contrast transfer function parameters. Bad movies were eliminated based on a CTF-fit resolution cut off at 5 Å and relative ice thickness of 1.2 estimated from the CTF function by cryoSPARC2. Different particle picking algorithms, including manual pick, template-based, and blob picking were employed to the same dataset and results on model distribution were compared. The evaluation of the density map at all stages and initial fitting of the Alphafold predicted model to the final density map were accomplished in Chimera [20]. The final structures were built and refined with program COOT [21]. Validation was done using Phenix [22]. The movies for the morphing between DNA-free and DNA-bound models were generated in ChimeraX [23].

AlphaFold structure predictions (Supplementary Table S2) were conducted using the ALPHAFOLD_PREDICT_COLAB tool embedded in ChimeraX [23].

#### Data collection and analyses of DNA-free enzyme

Cryo-EM grids were prepared by applying 2.5 μl of a SEC purified BsaXI at ∼0.3 mg/ml (ratio of OD^260^/OD^280^ at 0.57) to a glow-discharged Quantifoil1.2/1.3 holey carbon film coated copper grid, which was blotted for 7.0 s at a Tension of 10, and plunge-frozen in liquid ethane using an FEI Vitrobot Mark IV. Six screening datasets with a total of 2085 movies were collected on a FEI Glacios electron-microscope (operating at 200 kV) equipped with a Gatan K2-Summit direct electron detector at a pixel size of 1.16 Å. After three rounds of 2D classification/selection, 294 772 particles were used for a four-model 3D reconstruction. Only one of the four classes bore resemblance to the negative stained image and was refined to a resolution of 4.7 Å after homogenous, nonuniform and local refinements (Supplementary Fig. S1C).

A final full data set for the DNA-free BsaXI with a pixel size of 0.5395 Å was collected at PNCC using a Krios electron microscope (operating at 300 kV) equipped with a K3 direct electron detector. Following pre-processing, particle picking, 2D classification/selection, 3D reconstruction, and various refinements, the refined map at 3.26 Å resolution displayed a flexible hetero trimeric (RM)_2_S configuration with an asymmetrical S subunit manifested in different folds in the two TRD-CR domains (see Supplementary Fig. S2 for a flow chart of data processing and refinement).

#### Data collection and analyses of DNA-bound enzyme

The SEC purified BsaXI/DNA at an OD ^280^ of 1.56 (OD^260^/OD^280^ = 1.16) was used for grid preparation. The sample was diluted 3× with a high salt buffer containing 20 mM Tris–HCl (pH 8), 250 mM NaCl, and 2 mM CaCl_2_ to prevent aggregation. Cryo-EM grids were prepared with double blotting. Briefly, a 2.5 μl sample was applied to a glow-discharged Quantifoil1.2/1.3 holey carbon film coated copper grid which was clipped to the plunger in the Vitrobot chamber. The sample was allowed to adsorb to the grid for 10 s and the grid was blotted for 1 s to remove excess liquid followed immediately by the application of a second 2.5 μl sample. The second aliquot was allowed to adsorb for 2–3 s before blotted for 7 s and plunged into liquid nitrogen cooled ethane bath. Three full datasets were collected for the DNA-bound BsaXI. Two datasets with a total of 1579 movies were collected from two different grids at a super resolution of 0.56 Å pixel size using an in-house Glacios electron microscope operating at 200 kV and equipped with a K3 direct electron detector, and a third data set was collected at a super resolution pixel size of 0.5395 Å using a FEI Krios electron microscope at PNCC (operating at 300 kV and equipped with a K3 direct electron detector).

After preprocessing, reconstruction, and refinement separately to a resolution of 3.11 and 3.24 Å, respectively, the selected particles were reextracted and scaled together using an even box size of 330 pixel and Fourier crop factor determined by the python script boxscaler.py (Wilkinson, *et al.*, 2019). The final refinement using the combined particle stacks yielded a map of 3.06 Å at a GSFC of 0.143. 3D variability analysis showed that there were two different population of particles in the 3D landscape. Heterogeneous refinement separated the particle stacks into two almost evenly distributed classes with a third classes containing a small fraction of bad particles. The two different volumes were refined individually and yield two similar but clearly different maps that were individual fitted with unique models (see Supplementary Fig. S3 for a flow chart of data processing and refinement).

### Site-directed mutagenesis of BsaXI catalytic residues

To verify the identity and location of residues that are critical for DNA strand cleavage, fourteen residues (D57, D64, E71, E73, E75, D90, D95, D96, E105, K107, E114, K116, E118, and E120) that are located in the in the N-terminal endonuclease domain and seven additional residues (E811, K813, D816, E818, K820, E821, E823) that are located within and near to the tip of the double helix paddle were selected for point mutagenesis and subsequent activity assays. The mutagenic primers to generate Ala substitution for both mutagenesis experiments are listed in Supplementary Table S1.

PCR mutagenesis was carried out with the mutagenic primers (25 PCR cycles) and Phusion DNA polymerase. After spin column purification of the PCR products, the DNA was digested with DpnI to destroy the Dam^+^ template. Following heat inactivation of DpnI, the PCR DNA was treated with T4 polynucleotide kinase and Quick T4 DNA ligase at room temperature in quick ligase buffer for 30 min. The ligated DNA was transferred into C2566 competent cells and transformants were plated on LB agar plus Amp. Four individual colonies from each mutant were cultured in 2 ml LB + Amp overnight, and plasmids were prepared, and the entire gene was sequenced to confirm the mutated codon and absence of additional mutations.

The RM mutant plasmids were transferred into C2566 for small-scale expression and purification. 30 ml of mid-log phase cells were induced with IPTG (0.5 mM final concentration) and cultured at 18°C overnight. Cell pellets were resuspended in 2 ml of Ni-beads lysis buffer and lysed by sonication. Clarified lysates were mixed with Ni magnetic beads (100 μl beads suspension) for 1 h at 4°C on a roller. The Ni beads were washed 4 times with Ni wash buffer and the 8xHis tagged RM mutants were eluted with 100 μl of Ni elution buffer. 6xHis tagged BsaXI S subunit was purified from 1 l of IPTG-induced cells by DEAE column (flow through to remove nucleic acids) and Ni-agarose chromatography. For BsaXI complex enzyme reconstitution, 0.8 μg RM subunit (150 nM), and 0.4 μg S subunit (145 nM), nearly equal molar ratio of both subunits (previous mixing wild-type ('WT') RM and S subunits showed that adding excess amount of S subunit can slightly stimulate activity). The enzyme mix was used to digest 1 μg λ DNA in CutSmart buffer at 37°C for 1 h.

### Phylogenetic tree construction

94 individual sequences of Type IIB enzymes were aligned using MAFFT [24] to align the protein sequences only and then construct a tree using maximum-likelihood model with bootstrapping, or MAFFT_DASH [25] to integrate sequence and predicted structural topology information.

### Enzymatic cleavage assays with linear double-stranded DNA duplexes in various methylation states

DNA cleavage experiments were conducted on a series of four double strand DNA substrates, each containing either an unmethylated, a fully methylated, or each of two unique hemi-methylated target sites, flanked by sufficient base pairs to allow cleavage of either strand to either side of the target site.

To carry out this experiment, four fluorescently labeled oligonucleotides, corresponding to unmethylated or methylated top and bottom strands, were purchased from IDT. These single-stranded DNA (ssDNA) strands were of the following sequences (target site bases are underlined, adenine bases that are subject to methylation are bold):

- ***FAM_TOP_M6A:***
- 5′-FAM CAT GAA TAA GCT GAA TAT TGT CGG A**6mA**C CAA GTC TCC ATA TGG AAT TAA TAA GCT 3′
- ***FAM_TOP_REG (No modified adenine):***
- 5′-FAM CAT GAA TAA GCT GAA TAT TGT CGG A**A**C CAA GTC TCC ATA TGG AAT TAA TAA GCT 3′
- ***FAM_BOTTOM_M6A:***
- 5′-FAM AC TAT CGA CTA GCT TAT TAA TTC CAT ATG G6**mA**G ACT TGG TTC CGA CAA TAT TCA GCT TAT3’
- ***FAM_BOTTOM_REG (No modified adenine):***
- 5′-FAM AC TAT CGA CTA GCT TAT TAA TTC CAT ATG G**A**G ACT TGG TTC CGA CAA TAT TCA GCT TAT3’

The ssDNA oligos were annealed in each possible ‘top versus bottom strand’ pairwise combinations, to generate four double-stranded DNA (dsDNA) substrates in each possible methylation state. The various combinations of top and bottom strands were mixed in a 1:1 ration in Tris-EDTA ('TE') buffer (10 mM Tris–HCl, 0.1 mM EDTA) and heated at 95°C for 5 min in a PCR thermocycler and gradually cooled down to room temperature.

Annealed duplex oligos at 40 nanomolar final concentration were digested with 2 U of BsaXI (NEB R0609) in 1× NEB buffer 2 (50 mM NaCl, 10 mM Tris–HCl, 10 mM MgCl_2_, 1 mM DTT, pH 7.9) at 37°C for 30 min. The reactions were terminated by heat inactivation at 95°C for 10 min. Substrates and cleavage products were then resolved and quantitated by electrophoretic [capillary electrophoresis (CE)] analyses. The intact or digested oligos were run on the Applied Biosystems 3730xI DNA analyzer with GeneScan^TM^ 120 LIZ^TM^ dye Size Standard.

## Results

### Structure of DNA-free BsaXI

**Figure 1.**
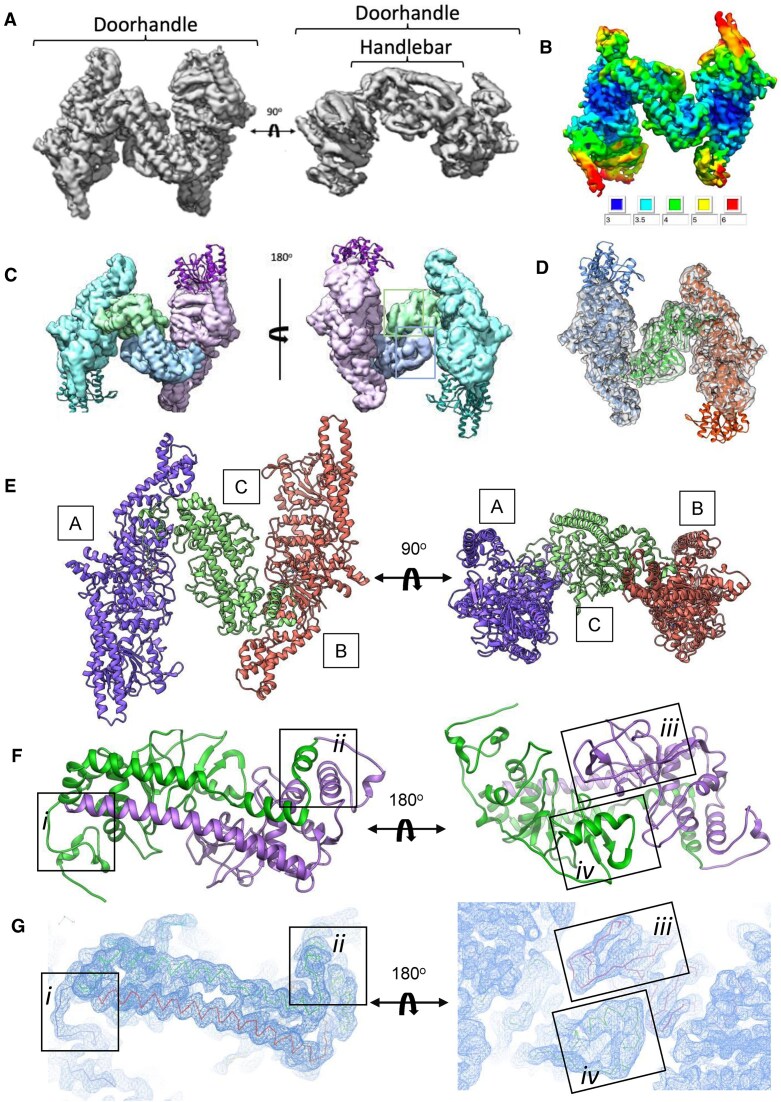
Structure of the DNA-free BsaXI enzyme assemblage. (**A**) Density map of DNA-free BsaXI at 3.2 Å resolution in two different orientations. (**B**) Local resolution of the density map ranging from better than 3 Å at the core to 6 Å at the periphery. (**C**) Segger segmentation map of apo-BsaXI in two different orientations, showing four contiguous sections corresponding to the two RM subunits (cyan and light purple) and the tip of the two TRD domains of the S subunit. (**D**) Overlay of the final apo-BsaXI model with the density map. The REase domains and the tip of the paddle domains are shown outside of the density at 7 sigma level. (**E**) Final ribbon model of the apo-BsaXI in two different orientations. In the corresponding PDB file, the RM subunits contacting TRD1 and TRD2 are assigned as chain A (purple) and chain B (orange), and the S subunit (green) is assigned as chain C. (**F**) Ribbon model of the S subunit showing TRD1-CR1 (green) and TRD2-CR2 (purple). (**G**) Density maps corresponding to the S subunit and its two TRD-CR domains.

**Figure 2.**
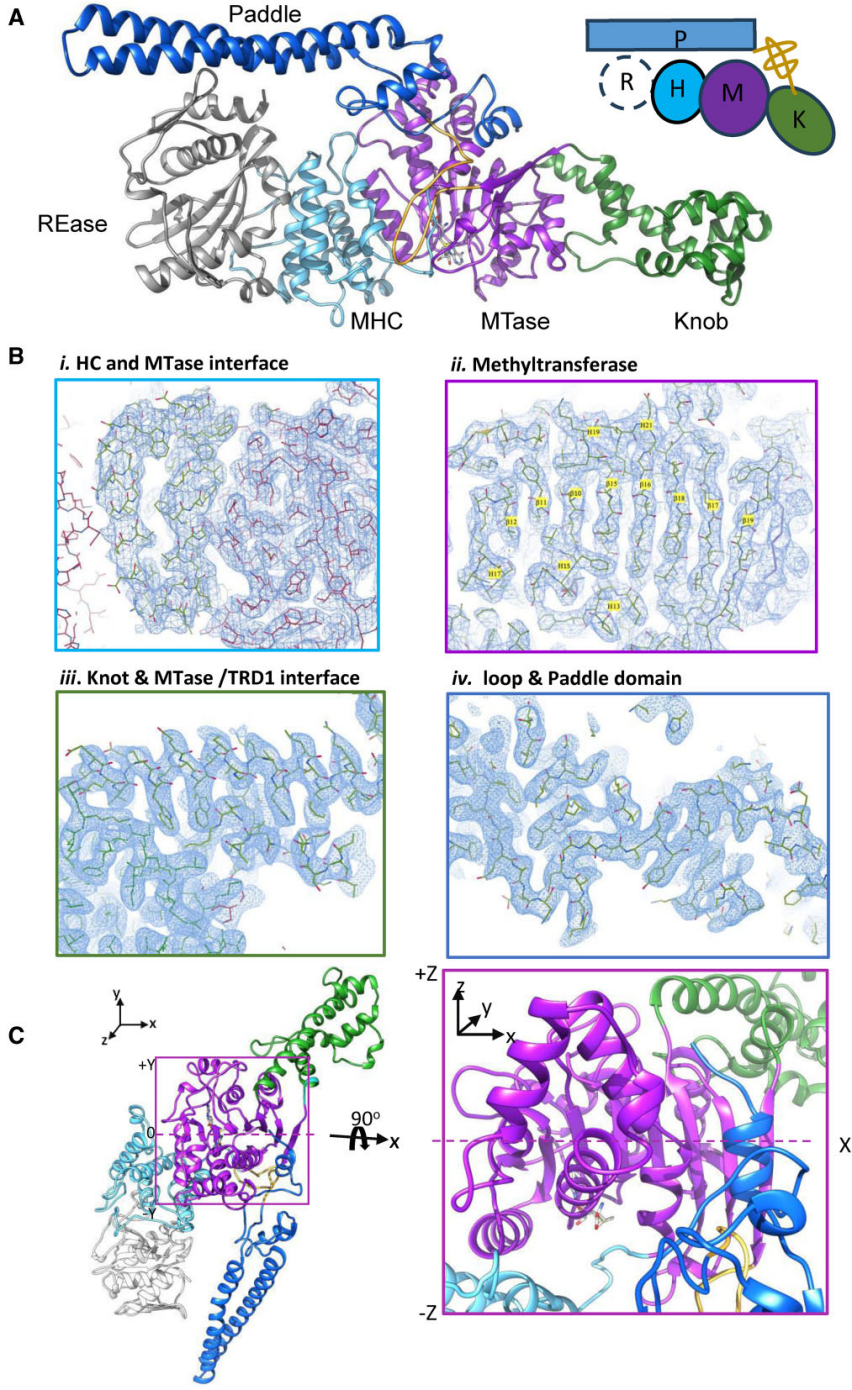
Details of the RM subunit of DNA-free BsaXI. (**A**) Ribbon diagram of RM subunit. Endonuclease: light gray; multiple-helix-connector domain: cyan; methyltransferase: purple; knob: forest green; loops: earth; and paddle: blue. Inset: Schematic representation of the BsaXI model. (**B**) Representative maps of different regions of the DNA-free enzyme. (**C**) Topographical schematic of the N^6^-adenosine methyltransferase domain with the central eight strand β-sheet in green and the five flanking helices in blue, solid outlined helices in the front, and dash-outlined ones in the back. The knob domain is inserted between the 7th and the 8th β-strands while the antiparallel double-helix paddle extends from the end of the 8th strand.

The refined cryo-EM map, at a global overall resolution of 3.3 Å (Table 1), indicated that the DNA-free BsaXI presents an ‘open’ conformation that resembles a door handle, with the S subunit occupying the position of the handlebar (Fig. 1A). Visualization of 3D variability (Supplementary Movie 1A and B), and local resolution analysis (Fig. 1B) showed that the particle is very flexible, particularly throughout the N-terminal endonuclease domain and across the tip of a long double-helical extension near the C-terminal end of each RM subunit (Figs 1C and D and 2A). A map in which the different molecular components of the protein are differentially colored and labeled for clarity (produced using the ‘Segger Segmentation’ plugin tool in UCSF Chimera visualization software [26]) illustrates four contiguous regions corresponding to the two RM subunits and two TRD of the S subunit (Fig. 1C).

**Table 1.** Cryo-EM data collection, refinement, and validation statistics of BsaXI

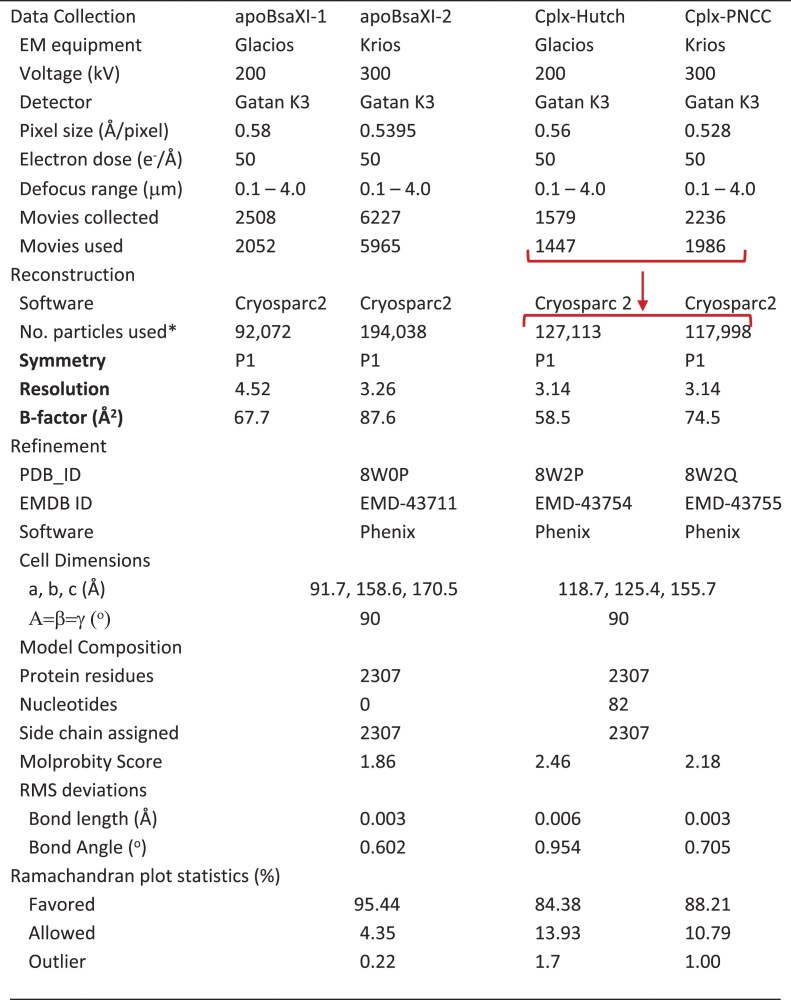

^a^Number of particles used for final refinement red brackets: particles combined from two data sets (up turned bracket) and, separated into two sets after heterogenous refinement (down turned bracket).

Our final refined model for the BsaXI, which was initially guided using models predicted by AlphaFold [27] and then further refined, corresponds to a heterotrimer displaying pseudo-2-fold symmetry with the two nearly identical RM subunits associated with either end of an elongated and asymmetric S subunit (Fig. 1E). The sections of the map corresponding to the RM subunits (Fig. 2) contain a well-defined multi-helical connector (residues 193–348, cyan) and a highly conserved mixed αβ fold characteristic of an N^6^-adenosine specific MTase domain (residues 363–625, purple), followed by two additional structure elements—a multi-helical domain with a central long helix wrapped around by four short helices at the solvent exposed end (residues 626–742; referred to hereafter as the ‘knob’ for its shape, green) which was inserted between strands β18 and β19 of the RM subunit and a long antiparallel two-helix bundle (referred to as the ‘paddle’ region, blue) that is stabilized by a twisted loop between its N- and C-terminus (Fig. 2 and Supplementary Fig. S5G). There was clear density corresponding to the cofactor SAM at the end of β-strand β15 in the MTase domain of each RM subunit (Fig. 2B, panel ii) corresponding to the end of β4 in the schematics of the conserved eight strand central β-sheet core of N^6^-adenosine methyltransferase (Supplementary Fig. S5).

The paddle region of BsaXI appears to represent a potentially novel structural element found within a RM enzyme. Results of a DALI similarity search [28] using the full-length BsaXI structure returned with an extensive list of hits against previously visualized N^6^-adenine methyltransferases and RM enzymes; however, none of the top Z score (Z > 20) hits contain this feature (Supplementary Fig. S5). The top score hits from a second Dali search using the paddle region as search template are mostly *de novo* helix bundle design proteins (data not shown). Hence it appears that naturally occurring proteins that present an entirely solvent-exposed, long antiparallel double helical motif resembling the paddle region in BsaXI are not highly represented in the PDB database.

The tips of the double helix Paddles appear to be quite flexible but could still be confidently placed and refined in the density at a lower contour level. The sporadic, poorly defined density surrounding the endonuclease (REase) domain in both RM subunits of the DNA-free structure indicated considerable dynamic flexibility and did not allow unambiguous placement of the domain. A model of that region, generated via AlphaFold [27], was placed at the strongest region of density within that region (Fig. 1C and D). The root mean square deviation (RMSD) between our final model of the RM subunits in the DNA-free BsaXI cryo-EM density and that predicted by AlphaFold was 1.33 and 1.36 Å for the A and B subunits, respectively.

The density corresponding to the S subunit can be divided into two regions, both corresponding to a globular target-binding domain (TBD) followed by a long helix formed by that subunit’s conserved region (CR). A pair of such helices (CR1 and CR2) form an antiparallel bundle (Fig. 1F and G), with a clear hinge point located halfway down the CR1 helix that forms a kink allowing CR1 and CR2 to rotate with respect to each other. The density of CR2 helix stops abruptly before reaching TBD1, while an additional helix connects the end of the TBD1–CR1 C-terminus and the N-terminus of the TBD2–CR2 (Fig. 1F and G).

**Figure 3.**
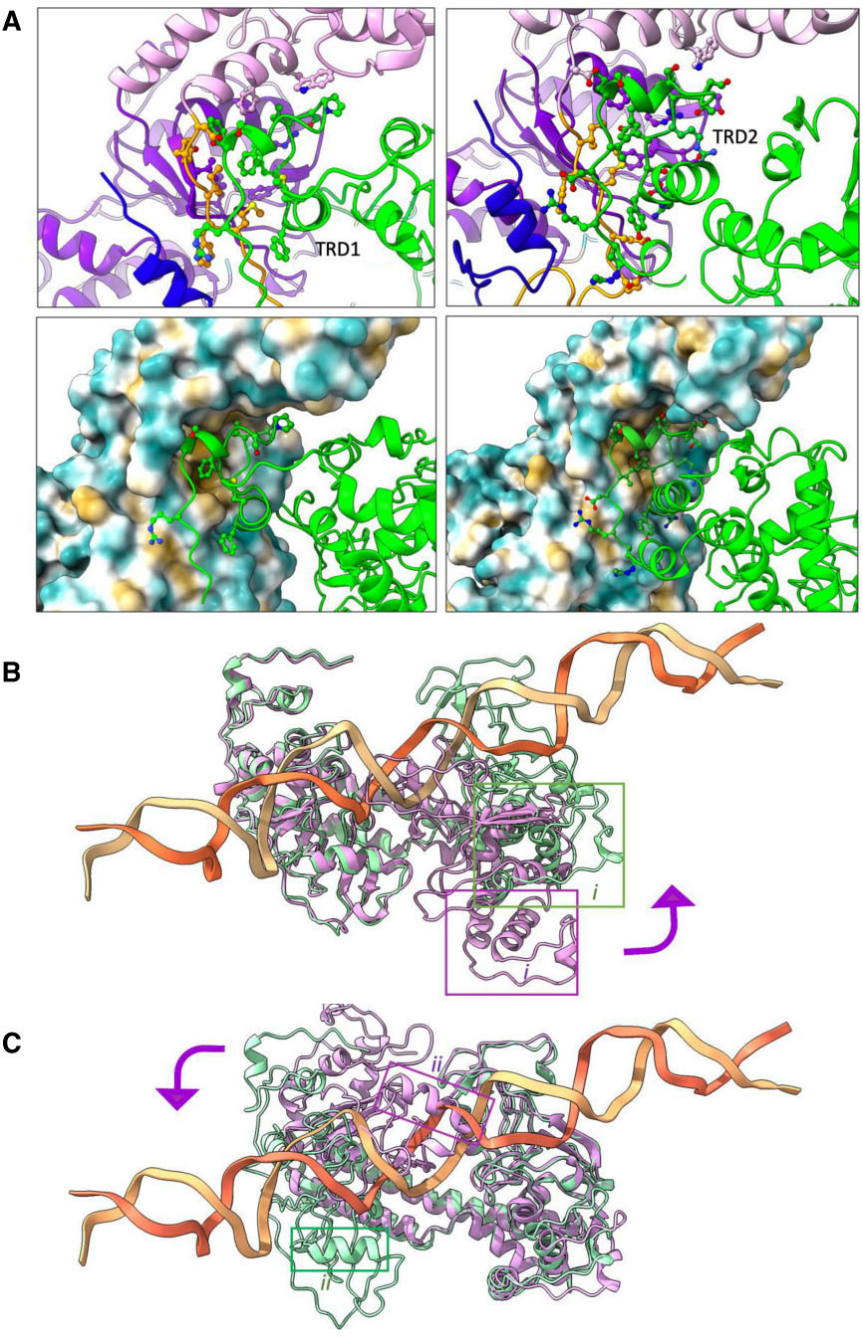
Intersubunit interactions and conformational changes in S subunit upon DNA binding. (A) Interactions between the RM subunits and the TRDs of the S subunit in the DNA-free BsaXI, showing the intercalation of the TRD domains into the hydrophobic pocket at the boundary of the MTase, loop, and knob domains. *Top panels*: The RM subunits are colored according to domains: MTase: dark purple; knob domain: light purple, loops: orange; paddle: blue; S subunit: green. *Bottom panels*: The surface of the RM subunits is colored according to hydrophobicity of the surface residues. (**B, C**) Superposition of the DNA-free BsaXI S subunit (purple) with the S subunit of the DNA-bound (green) showing different rotation of the TBD domains in the two conformers upon binding to the bipartite DNA target. (**B**) Superposition of S subunits from DNA-free BsaXI and DNA-bound conformer I show close alignment of TBD1 domain and CCW rotation of the TBD2 domain (purple curved arrow). (**C**) Superposition of S subunits from DNA-free BsaXI to DNA-bound conformer II show close alignment of TBD2 and CCW rotation of TBD1.

While the refined models for the two RM subunits are extremely similar, the two TRD domains in the S subunit differ significantly from each other, corresponding to an RMSD of 3.35 Å across 167 superimposed alpha carbons from the two TRDs. There are no significant contacts formed between the two RM subunits in the DNA-free BsaXI structure, and only a small contact surface area is observed between each RM subunit and one of the two TBDs of the S subunit. Both TRD domains are tucked into a hydrophobic cradle formed by the base of the RM subunit’s knob domain and the 8th β-strand of the central β-sheet of the MTase domain (Fig. 3). The buried surface area between the two RM subunits and the S subunit is ∼1800 Å for each TRD, respectively.

### Structures of DNA-bound BsaXI

**Figure 4.**
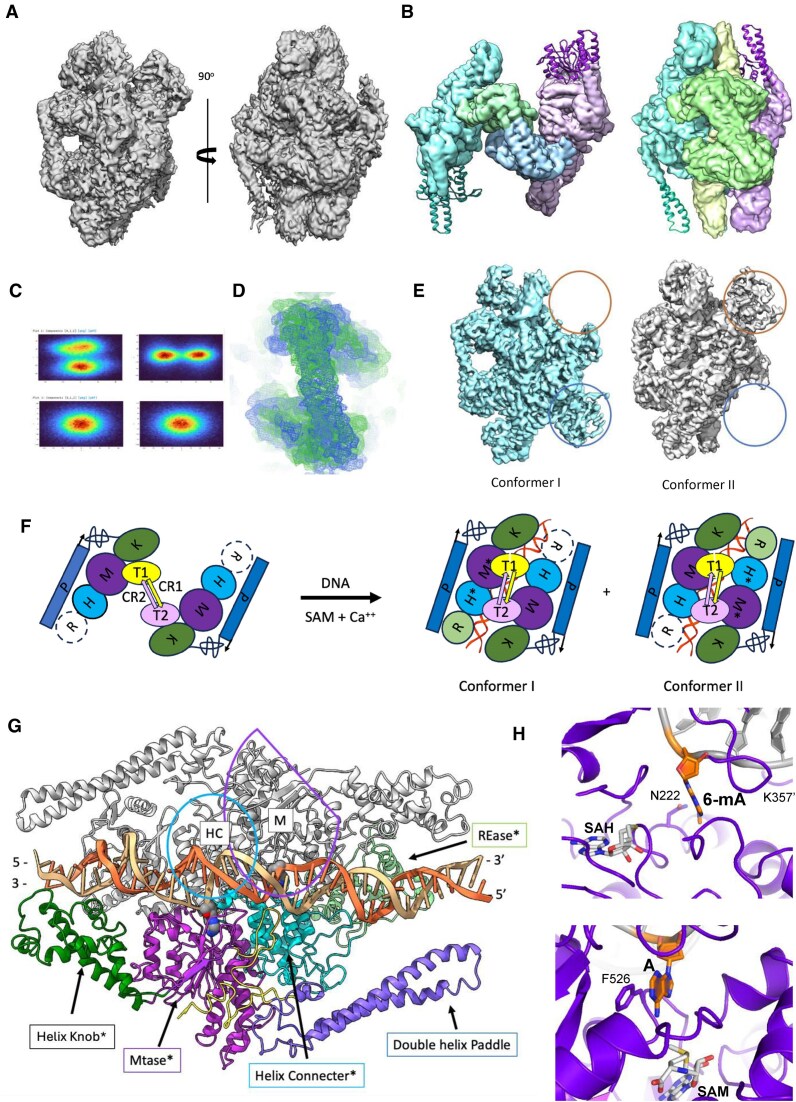
Cryo-EM structures of DNA-bound BsaXI. (**A**) Two different orientations of the initial 3D volume for BsaXI–DNA particle, showing the “Closed” conformation, the long handlebar configuration of the S-subunit, and the supercoil double helix of the DNA. (**B**) *Left*: Cryo-EM map of DNA-free BsaXI overlaid with ribbon representation the (RM)_2_S protein structure. *Right*: Map if the BsaXI/DNA complex (overlaid with the ribbon representation of the initial model for the DNA-bound conformation of the (RM)_2_S-DNA complex. (**C**) 3D variability analysis of the DNA-bound enzyme complex, showing the presence of two populations of particles before and after heterogeneous refinement (top and bottom, respectively). (**D**) Superposition of the maps corresponding to the S-subunits in the two DNA-bound conformation after heterogenous refinement (blue and green), versus the initial map (gray), showing different disposition of the TRDs. (**E**) Comparison of the refined maps of the two different DNA-bound enzyme conformations. Orange and blue circles highlight the presence and absence of density for the REase domain. (**F**) Schematic presentation of conversion from DNA-free to DNA-bound BsaXI particles deduced from cryo-EM maps. The endonuclease domain (‘R’) visible at one of two positions in each DNA-bound conformation is indicated by a highlighted circle at each position. (**G**) Domain organization of BsaXI–DNA complex with the S-subunit removed from the top layer, showing a multiple-colored ribbon model of RM* subunit A, a ribbon model of RM subunit B in gray, and the double-stranded DNA. The top DNA strand is beige, and the compliment strand is orange. The upstream AC target is on the left and CTCC target on the right. The RM* subunit that interacts with the flipped-out bases is depicted in multicolor ribbon. The nuclease domain REase* is pea green, followed by the multiple helical connector* (cyan), MTase* (purple), the knob* (forest green), and the Paddle (blue). The long antiparallel double helix Paddle domain (corn flower blue) is tethered to the knob region by a network of loops (yellow). The upstream AC target is on the left with the flipped-out adenine base (space filling) inserts into the MTase* domain. The N^6^-methyl-adenine nucleotide (space filling) in the complement to the downstream (CTCC) target is on the right pointing toward the multi-helical connector* (HC*; cyan ribbon). The other RM subunit (light gray) is antiparallel to chain A with the MTase (M**)** domain (purple outline) directly above the multi-helix connecter* (cyan ribbon); the multi-helix connecter (cyan outline) of the other subunit is positioned against the MTase* domain of the RM* subunit. (**H**) Close-up views of the two adenine bases [one in the 5′ AC target region (*upper panel*) and the second in the opposing strands of the 3′ CTCC target region (*lower panel*)] that are each flipped out of the DNA duplex and located in binding pockets formed by one or both RM subunits, and targeted for methylation. The former base is methylated, while the second is unmethylated; they are located proximal to bound S-adenosyl-L-homocysteine (SAH) and SAM cofactor molecules that are observed in each of the two respective MTase active sites.

Initially, all selected particles were used to generate a single electron density map with global resolution of 3.05 Å (Supplementary Fig. S3C–I, volume iii). It clearly showed that upon binding to its cognate DNA target, the “open” conformation of the DNA-free BsaXI described above undergoes a large conformational change leading to a “closed” conformation with the three subunits wrapping around a bent DNA duplex at the center. The handlebar configuration of the S subunit is evident on one side of the particle, and the MTase domains of the two RM subunits are in close contact on the opposite side of the complex (Fig. 4, Supplementary Fig. S4, and Supplementary Movie 2).

#### BsaXI samples two closely related DNA-bound conformations

Modeling and refinement of a single DNA-bound enzyme complex into the map described above was unexpectedly challenging, especially near the ends of the antiparallel helices of the S subunit and the underside (DNA-contacting) regions of that same subunit’s TBD domains. Closer inspection indicated that the density footprints spanning each of the TBD domains were significant larger in the DNA-bound complex than in the DNA-free enzyme (Fig. 4B). This suggested that the refined 3D map of the BsaXI–DNA might represent a composite of multiple DNA-bound conformations. A subsequent 3D variability analysis of the initial map showed that there were indeed two significant populations of particles in the 3D landscape (Fig. 4C). Further heterogeneous refinement using the initial ∼249k particles showed that the initial map represented a superposition of two unique electron density maps that differed in the orientation of the S subunit (Fig. 4D) with respect to the spatial disposition of the single REase domain (Fig. 4E and F).

**Figure 5.**
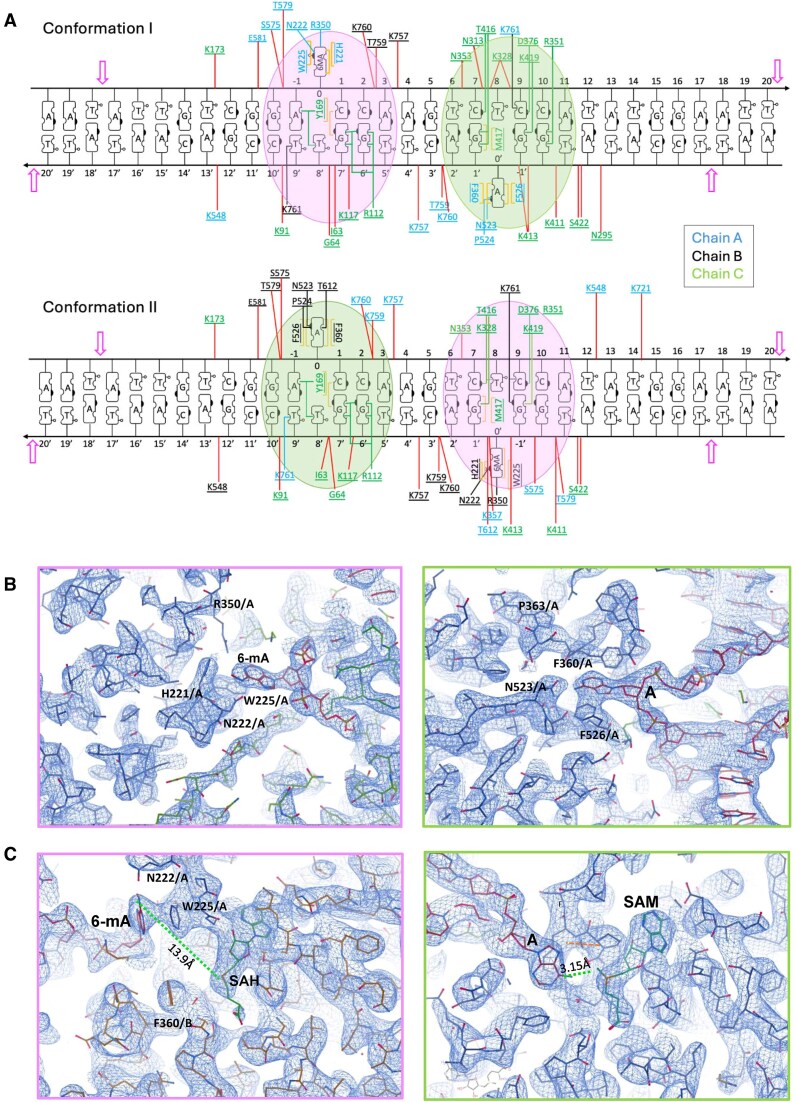
Protein–DNA interactions in the two conformations of the DNA-bound BsaXI complex. (**A**) Direct contacts made by residues from chains A (RM subunit 1), B (RM subunit 2), and C (S subunit) are marked in black, blue, and green, respectively. The four magenta open arrows at the ends indicate the position of the scissile phosphodiester linkages. The red lines indicated nonspecific hydrophilic (electrostatic) interactions between the rotamer and/or peptide backbone of proteins and the DNA phosphate back bone. The numbering next to the phosphate backbone indicate the position of the base pairs relative to the flipped-out adenine or 6MA. For clarity, only some of the nonspecific electrostatic interactions are indicated. The green and pink filled circles highlighted the surrounding of the flipped-out adenine and the N^6^-methylated-adenine (6MA) in the two conformers. The S subunit utilizes identical residues in direct contacts with the DNA target in both bound conformations. Two residues (Y169 and R112) in the S subunit contact the upstream AC target site and five residues (R351, D376, T416, M417, and K419) contact the downstream CTCC target site. Almost all contacts between an RM subunit and DNA are exclusively made by one or the other subunit in the separate bound conformations. The flipped-out adenine is stabilized by F356, N523, P524, F526, and T612 from the MTase* domain. The flipped-out 6MA is surrounded and stabilized by four residues (R350, H221, N222, and W225) from the multi-helical connector domain. (**B, C**) Density features surrounding the flipped-out adenine (left) and N^6^-methylated-adenine (right).

Model building into each of the two individually refined maps, respectively corresponding to two individual DNA-bound BsaXI conformations (Table 1), was then straightforward. Each conformation of the BsaXI–DNA complex places the two RM subunits in an antiparallel arrangement, on either side of the S subunit and the DNA duplex (see Fig. 4G for a ribbon representation of conformer I). The multi-helical connector region and MTase domains on both RM subunits, together with the S subunit, are wrapped tightly around the central 11 base-pairs encompassing the entire sequence of the bipartite 5′-**AC**NNNNN**CTCC**-3′ target site (and its complement) plus one base pair before the upstream AC site (Fig. 5).

The two TRDs of the S subunit bind to the two half-sites in the bipartite DNA target in only one orientation. The TBD1 domain is bound to the base pairs corresponding to the upstream 5′-AC-3′ sequence, while the TBD2 is bound to the base pairs corresponding to the downstream 5′-CTCC-3′ sequence. Almost all direct contacts to the target site are contributed by the S-subunit (Fig. 5A and Supplementary Fig. S6), which largely employs identical residues to contact the target in the two independently refined conformations of the DNA-bound enzyme.

In each map, clear density was observed for only one of the REase domains, while the second REase domain on the opposing RM subunit (i.e. at the opposite end of the DNA target) was too disordered to be modeled. Thus, binding of BsaXI to its bipartite target triggers a large conformational closure around the DNA, that in-turn creates a nearly equal distribution of particles differing in the spatial ordering of a single REase domain in each structure (Fig. 4E and F). The difference between these two conformations can be analogized as similar to a playground ‘teeter-totter’, with the two DNA-bound states displaying different contacts and distances between the individual protein residues and the nucleotides within the two halves of the bipartite DNA target site (described in the next section). Because of the similarities in the structure of the two conformations, detailed descriptions of only one conformation (specifically conformer I) are presented.

#### An adenine base in each strand is flipped out and appears to be targeted for methylation

Density maps of both conformers clearly showed that two adenine bases (one in the 5′ AC target region, and a second in the complement of the 3′ CTCC target region) are simultaneously flipped out from the center of the double-strand DNA. Each is captured in a unique binding pocket near the methyltransferase active site and proximal to a bound SAM (or SAH) cofactor (Fig. 5B and C). The adenine base in the 5′ AC target region of conformer I is observed to be methylated (i.e. converted to 6-methyladenine) and is located in a pocket formed within an interface between the MTase domains of the two RM subunits, where it interacts with a demethylated SAH cofactor. In contrast, the adenine base in the 3′ target CTCC region of conformer I (in the complementary DNA strand) is unmethylated and is located in a pocket formed solely within the MTase domain of a single RM subunit, where it interacts with a bound SAM cofactor (Figs 4H, and 5B and C, and Supplementary Fig. S7B and C).

#### Direct protein–DNA interactions

All but one of the residues involved in direct contact with the DNA in both conformers are from the S-subunit (Fig. 5A and Supplementary Fig. S6B). Y169 of the TBD1 is inserted into the space evacuated by the flipped-out adenosine (A0) in the upstream AC site and interacts with the second upstream target pair (C1:G7’) via π-stacking, while the hydroxyl group interacts with the orphaned thymine (T8’) and the upstream adenosine (A-1). The guanidium group of Arg112 in TBD1 forms multiple contact with the complement guanine (G7’) of the second upstream C:G target pair and a purine (G6’) in the complement strand of the variable sequence.

At the downstream 3′ CTCC half-site, M417 of TBD2 is partially inserted into the space evacuated by the flipped-out base and π-stacked with the 3′-guanine (G1’) of the complement strand. The interactions between TBD2 and the first downstream target C:G pair (C7:G1’) were reinforced by H-bonds with T416 of TBD2. While interactions of TBD2 with the lone thymine (T8) and the two downstream C:G pairs were provided by multiple contacts with R351 and K419. In addition, N313, K328, N353, K411, K413, and K422 of the TBD2 also form electrostatic interactions with the phosphodiester backbone near the downstream CTCC target site.

In both conformers, the flipped-out unmethylated adenine base is stabilized by π-stacking with F360 and F526 and by H-bonds with N523, P524, and T612, all from the MTase domain (Figs 4H and 5B, left). The flipped-out, methylated N^6^-methyl-adenine (6MA) is similarly captured by π-stacking with H221 and W225 and by contacts with N222 and R350 [residues located in the multi-helical connector (Figs 4H and 5B, right)]. There is only one residue (K761 in each RM subunit) that is involved in direct contact with DNA (specifically, T9’) in both conformers. Finally, the sparse direct contacts between the RM subunits and the target DNA is reinforced in both conformers, by positively charged β-hairpins between the lysine-rich β20-turn-β21 (residues 755–768) that wrap around the bipartite DNA target and form multiple electrostatic interactions with the phosphate backbones at the variable bases in between the two sites.

### Transition between DNA-free and DNA-bound conformations

Conformational analyses of the enzyme-bound DNA superimposed on an ideal model of B-form DNA reveals that binding of BsaXI resulted in bending and widening of the double strands (Supplementary Fig. S7D and E), as well as an ∼10% reduction (from 34.3 to 30.4 Å) in the distance between the two adenine bases, one in each half site of the bipartite target, and that the configurations of the 5 variable base-pairs insert between the two half-sites are least deviated from that of idea-B-form DNA.

Docking the coordinates of an ideal B-form DNA via LSQ superposition with that of the five variable inserted base pairs followed by SSM superposition of the RM subunits of the DNA-free BsaXI to the respective RM* subunits in the DNA-bound complexes allowed us to model two hypothetical ‘initial encounter’ models: one with the upstream AC sequence closer to TRD1, versus a second with the downstream CTCC (and its complement GAGG) half-site closer to TRD2 (Supplementary Fig. S8A and B). Movies from morphing of the hypothetical “initial encounters” and the DNA-bound conformations using the program ChimeraX [23] suggested that during the transition from the Open DNA-free to the Closed DNA-bound configuration, the position of one RM subunit in the DNA-free BsaXI (i.e. the RM* subunit), with the exception of the flipping and swinging REase, remains relatively stationary, while the second RM subunit moves from an open extended arm into proximity with the DNA (Supplementary Movie 3A and B).

#### DNA cleavage sites

**Figure 6.**
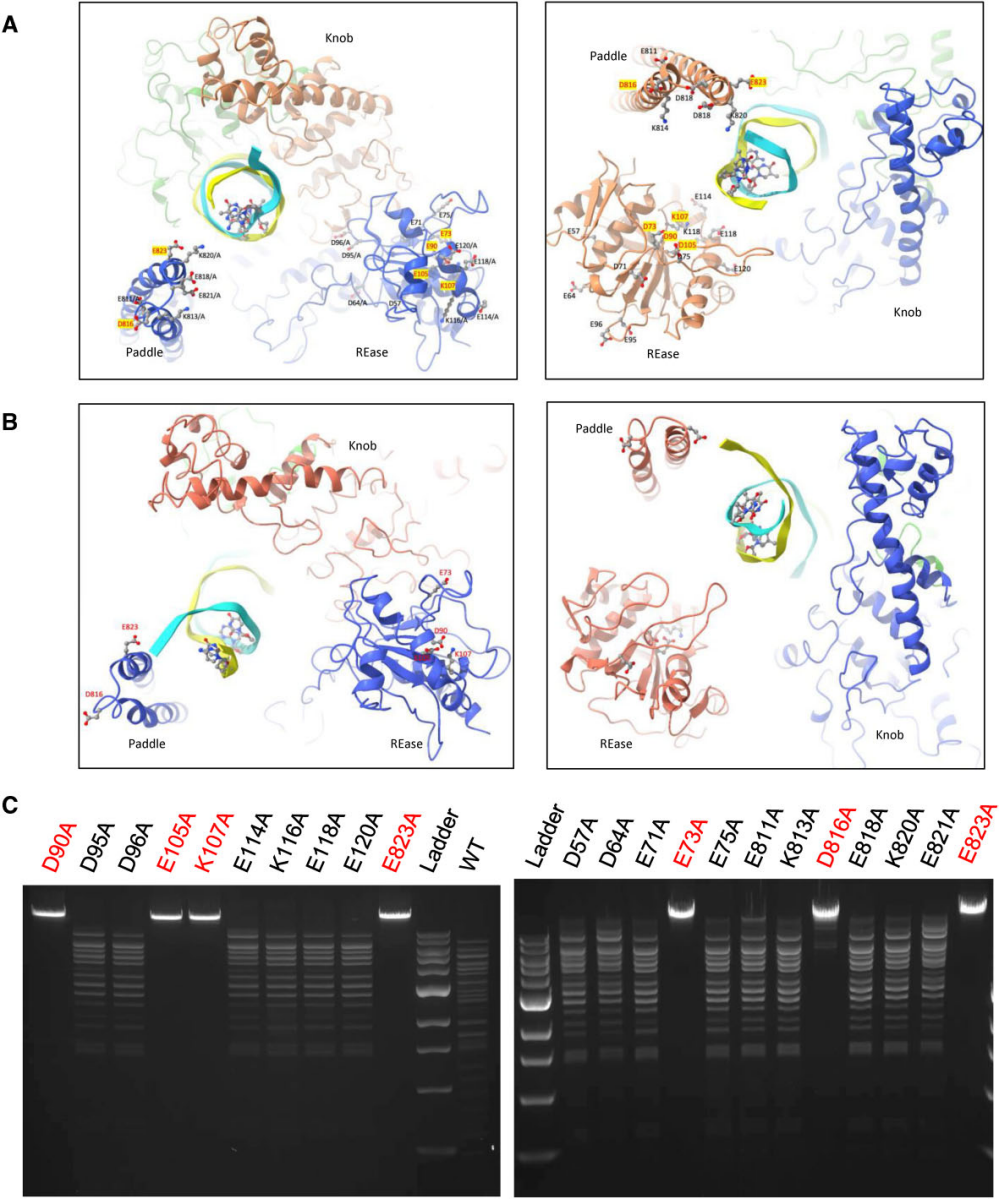
Mutational effect of alanine substitution at residues in the N-terminal endonuclease domain and C-terminal paddle domain. (**A**) Two views, from different perspectives, of the interaction of a portion of an RM subunit near one of the two DNA cleavage sites (upstream of the 5′ AC 3′ target region), showing the proximity of the N-terminal endonuclease domain (REase) and C-terminal paddle region to the two DNA strands near the cleavage site. (**B**) Residues in the REase domain and paddle region that were individually subjected to single-site alanine point mutagenesis. Those positions that resulted in the inactivation of DNA cleavage activity upon mutagenesis are indicated in red font. (**C**) Phage λ DNA (which contains 19 BsaXI target sites and runs as a single linear species near the top of the gel in the absence of enzyme) was digested with WT reconstituted BsaXI (leading to complete digestion and the appearance of multiple DNA fragments of reduced size and increased electrophoretic mobility; right side of lower gel) and with similar concentrations of a panel of individual alanine point mutants placed at individual positions in the endonuclease domain (D57A through E120A) and the paddle region (D811A through E823A). Mutations at four positions in the endonuclease domain (E73A, D90A, E105A, and K107A) and two positions in the paddle region (D816A and E823A) cause obvious, complete inactivation of the enzyme. Additional cleavage experiments using plasmid substrates (not shown) show no indication of nickase activity above background, indicating that DNA cleavage appears to be fully suppressed by these mutations. (Note that D816A mutant still has a low partial activity.)

BsaXI generates two double-strand breaks, one on either side of the bound target site. The sites of DNA strand scission correspond to scissile phosphate groups located 9/12 and 10/7 bases up- and down-stream of the target site on the top and bottom DNA strands, symmetrically 10 and 20 base-pairs up- and down-stream, respectively, from the flipped-out base in either strand. Thus, the enzyme generates four separate strand breaks and a pair of precisely defined, 3 base, 3′ overhangs at each cleavage site. The N-terminal REase domains (residues 1 to 192), and C-terminal double-helical ‘paddle’ regions (residues 800 to 835) (Figs 4G and H, and 6A and B) are located near the respective sites of each double strand break, but neither region of the enzyme is observed in an intimately bound, cleavage-competent complex with the scissile phosphates at those sites. In each of the two separately refined conformations of the DNA-bound enzyme complex, only one of the two REase domains is clearly visible, while the other is disordered (Fig. 4F). In contrast, in both DNA-bound-structures both ‘paddle’ regions (Fig. 4G) are clearly observable.

The same 3D variability analyses described above, which led to the observation and modeling of two closely related DNA-bound complexes, also indicated that one of the two nuclease domains (REase domain 1) appears more flexible than its counterpart (Supplementary Movie 4A and B). Its density level varies from nearly unobservable to almost fully covering the model in both conformations, whereas the electron density corresponding to REase domain 2 is fully resolved in conformer I, but absent in conformer II.

### Activity of enzymatic point mutants

Due to the presence of multiple putative PD-(D/E)xK motifs in the endonuclease domain of the RM subunit, alanine substitution mutagenesis was performed at fourteen positions in the N-terminal endonuclease (REase) domain (D57, D64, E71, E73, E75, D90, D95, D96, E105, K107, E114, K116, E118, and E120). In addition, another seven positions in the C-terminal paddle region that were also proximal to the cleavage sites (E811, K813, D816, E818, K820, E821, and E823) were also subjected to alanine substitutions (Fig. 6A and B). All twenty-one constructs were expressed and remained soluble and provided similar proteins yields from affinity purification columns. Of these point mutants, mutations at four positions in the REase domain (E73, D90, E105, and K107) and two positions in the C-terminal paddle region (D816 and E823) led to obvious inactivation of cleavage activity (Fig. 6B). However, none of the six knockout mutants, in the REase domain or in the paddle, exhibit obvious nickase activity.

### The effect of methylation and hemi-methylation on DNA cleavage

**Figure 7.**
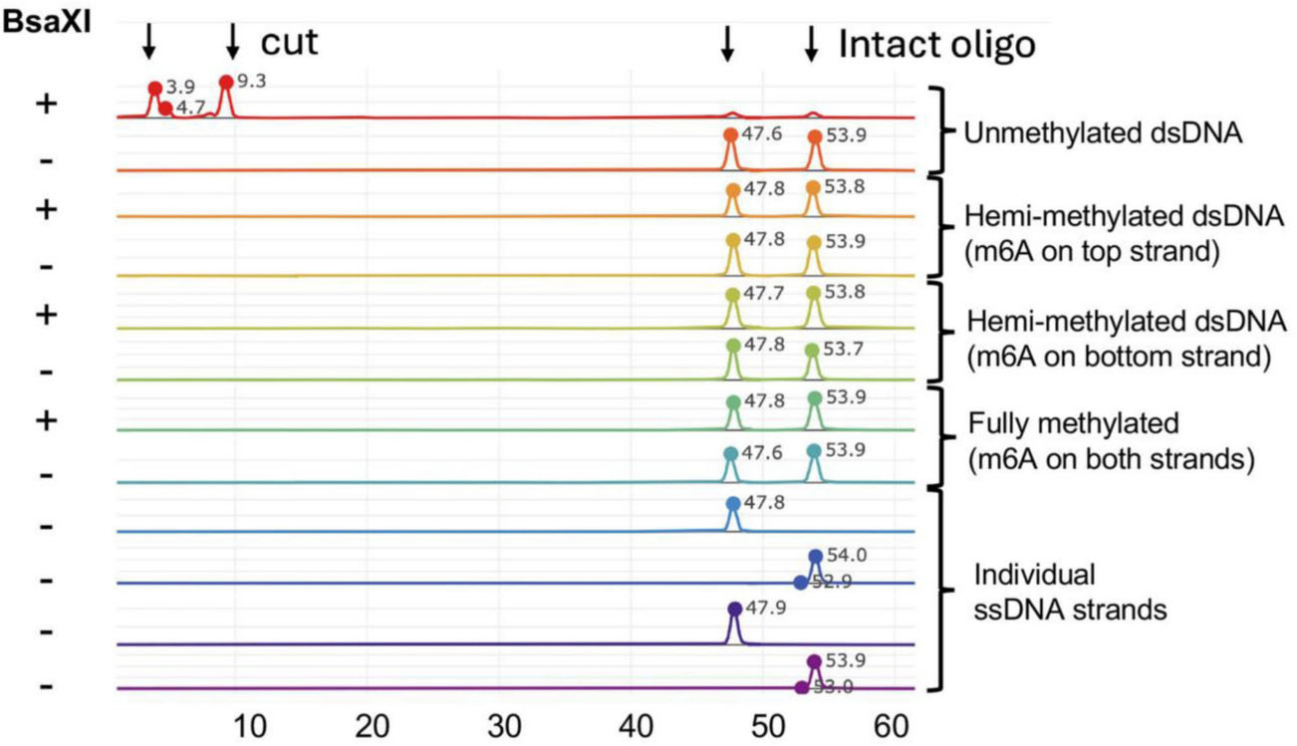
Hemimethylation on either target strand is sufficient to prevent DNA cleavage to either side of the enzyme’s target site. Four separate fluorescently labeled dsDNA duplexes (each containing a central bipartite target site for BsaXI, in each of four possible methylation states) were digested with BsaXI as described in the ‘Materials and methods’ section, and the resulting digest products were visualized and quantitated using CE analysis. While the unmethylated dsDNA substrate is cleaved to completion to either side of the target site, both hemi-methylated substrates and the fully bi-methylated substrates show no sign of cleavage in this experiment.

We went on to assess the effect of methylation at either (or both) of the two adenine bases described above (that are each flipped out in the DNA-bound enzyme complexes and targeted for methylation) on DNA cleavage at either side of the bound target site (Fig. 7). Cleavage digests using four separated dsDNA substrates (individually harboring fully unmethylated, fully methylated, or each of the two possible hemi-methylated target sites) demonstrated that only the fully unmethylated target site could be cleaved by the enzyme.

## Discussion

### BsaXI is an addition to recent structural studies of complex multi-domain RM systems

Several hundred high resolution crystallographic structures have now been determined of stand-alone Type II enzymes, as well as various subunits and assemblages corresponding to more complex Type I, III, and IV systems. The recent advent of high-resolution cryo-EM has greatly facilitated the visualization of highly dynamic assemblages of RM enzymes that previously confounded efforts for crystallographic analyses. Noteworthy recent structural studies include:

- Crystallographic structures of two Type ISP enzymes (LlaGI and LlaBIII), that incorporate a RecA-like ATPase into their single chain architectures and rely on that domain to drive locomotion from separate bound sites and eventual DNA cleavage [29, 30];
- Crystallographic structures of two Type IIG(L) enzymes (BpuSI and MmeI) that correspond to a Type II single-chain RMS configuration [31, 32];
- Crystallographic and cryo-EM structures of a DNA-free and DNA-bound Type IIS enzyme, PaqCI, demonstrating significant conformational rearrangements leading to DNA cleavage [33];
- Cryo-EM structures of the DNA-bound form of Type IIG(S) enzyme DrdV in various assemblage states involving two, three, or four DNA-bound enzyme subunits [34];
- A crystallographic structure of a Type III multichain EcoP15I complex with two MTase and a REase subunits that bound to DNA [35];
- A crystallographic structure of a Type IV methyl-dependent restriction endonuclease, MspJI, in a tetrameric complex of MspJI bound to DNA [36, 37];
- Cryo-EM structures of the Type I enzyme EcoR124I (a multichain complex containing multiple nuclease-translocase, methyltransferase, and specificity subunits) bound to DNA [38] (see Supplementary Fig. S8). This analysis expanded upon lower-resolution models of DNA-bound ‘M_2_S’ subcomplexes of that same enzyme [39], as well as structures of EcoKI [40] and of TteI [41].

Collectively, these analyses have provided considerable insight into the domain organization, structural dynamics, DNA recognition specificity, a unique mechanism of translocation and subsequent cleavage of DNA exhibited by Type ISP systems [29, 30] and suggestions of how multimeric RM enzyme ensure protection of self- versus degradation of foreign DNA [33, 34]. However, comparative high-resolution structures of a multimeric RM(S) enzyme system in both a DNA-free and a DNA-bound forms, which would illustrate the transition from one state to the other, have not yet been available.

### Structural modeling of additional type IIB enzymes

**Figure 8.**
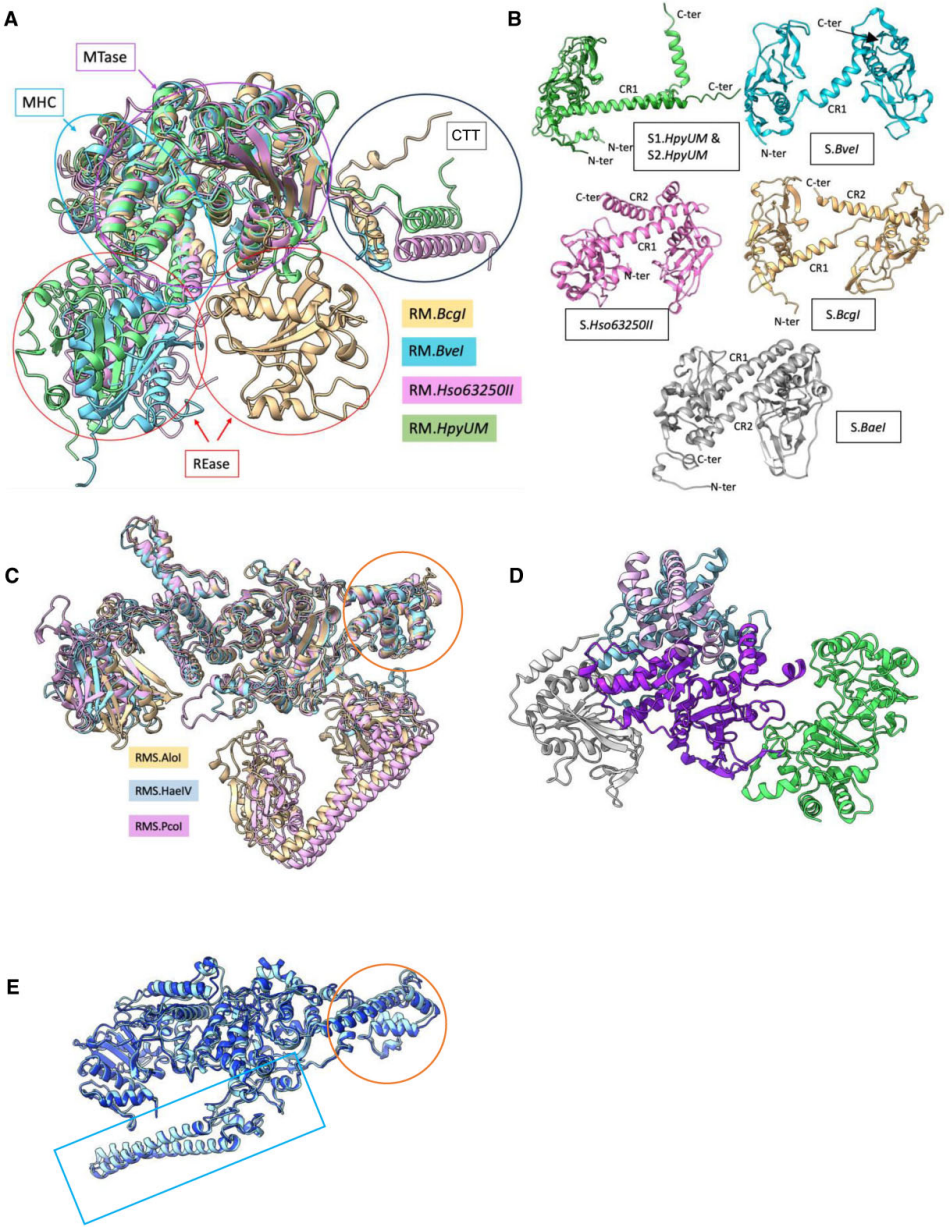
Summary of AlphaFold structure predictions for identified Type IIB RM systems. (**A**) AlphaFold models of Type IIB enzyme homologues with Type I-like RM/S subunit compositions. *Left panel*: Overlay of predicted models for RM fusion of *BcgI* (badge), *BveI* (cyan), *Hso63250II* (pink) and *HpyUM* (green) showing the excellent superposition of all R (REase), H (multi-helical connector; MHC), and M (MTase) domains except the REase of *Bcg*I and the very flexible C-terminal tails. *Right panel top*: Predicted model for the S subunit of *BveI* showing the TRDs and antiparallel CR1 and CR2. *Right panel bottom*: predicted model for the S subunit of *BcgI* with skewed CR1 and CR2. The distorted arrangements of the two TBDs and CRs in the skewed S subunit could be the reason why *BcgI* requires higher order arrangements for activity. (**B**) Overlay of examples of predicted models of Type IIB RM system with a single-chain RMS composition, indicating the presence of the knob domain (orange circle) and the characteristic (TRD1–CR1–TRD2–CR2) fold of the Type I HsdS subunit. The latter is tethered to the MTase domain via a flexible loop. (**C**) Superposition of the predicted model and the cryo-EM structure of BsaXI (light and dark blue, respectively). All the predicted models of the identified Type IIB with single chain chimeric RMS configuration have a knob domain (orange circle), but BsaXI is the only Type IIB with both the knob (orange circle) and the long double helix-paddle (cyan circle) domains as observed in the cryo-EM structure. (**D**) Predicted structure of RdeGBIII, the Type IIB RMS fusion peptide with the S domain resembles that of the Type IIG proteins BpuSI and MmeI. (**E**) X-ray crystal structure of BpuSI.

There are a total of 28 identified Type IIB enzymes in the REBASE database [42], of which the sequence of 16 were available and obtained via download or from their original discoverers. Results from AlphaFold structure [27] predictions for all 16 systems (Fig. 8 and Supplementary Table S2) indicated that except for three that displayed partial or poorly modeled folds (BceSIV, NgoAVIII, and NmeDI), their predicted structures can be readily separated into two groups. Eight of the sixteen predicted structures, including BsaXI, are encoded as single RM fusion proteins and a separate S subunit (RM + S group), while the remaining proteins form single-chain RMS fusion proteins. Among the ‘RM + S’ group, all the MTase domains, except for BsaXI, display a fold similar to the Type I methylase M.EcoKI, with a lone helical tail at the protein’s C-terminus that was previously shown to form four helix bundle with the CR1 and CR2 of the substrate recognizing subunit in the M_2_S complex (Fig. 8A). It is worth noting that all but one (i.e. *S.BaeI*) of the AlphaFold predict model of the identified Type IIB S-subunits display either incomplete or askew TRD1–CR1–TRD2–CR2 modeled folds (Fig. 8B).

**Figure 9.**
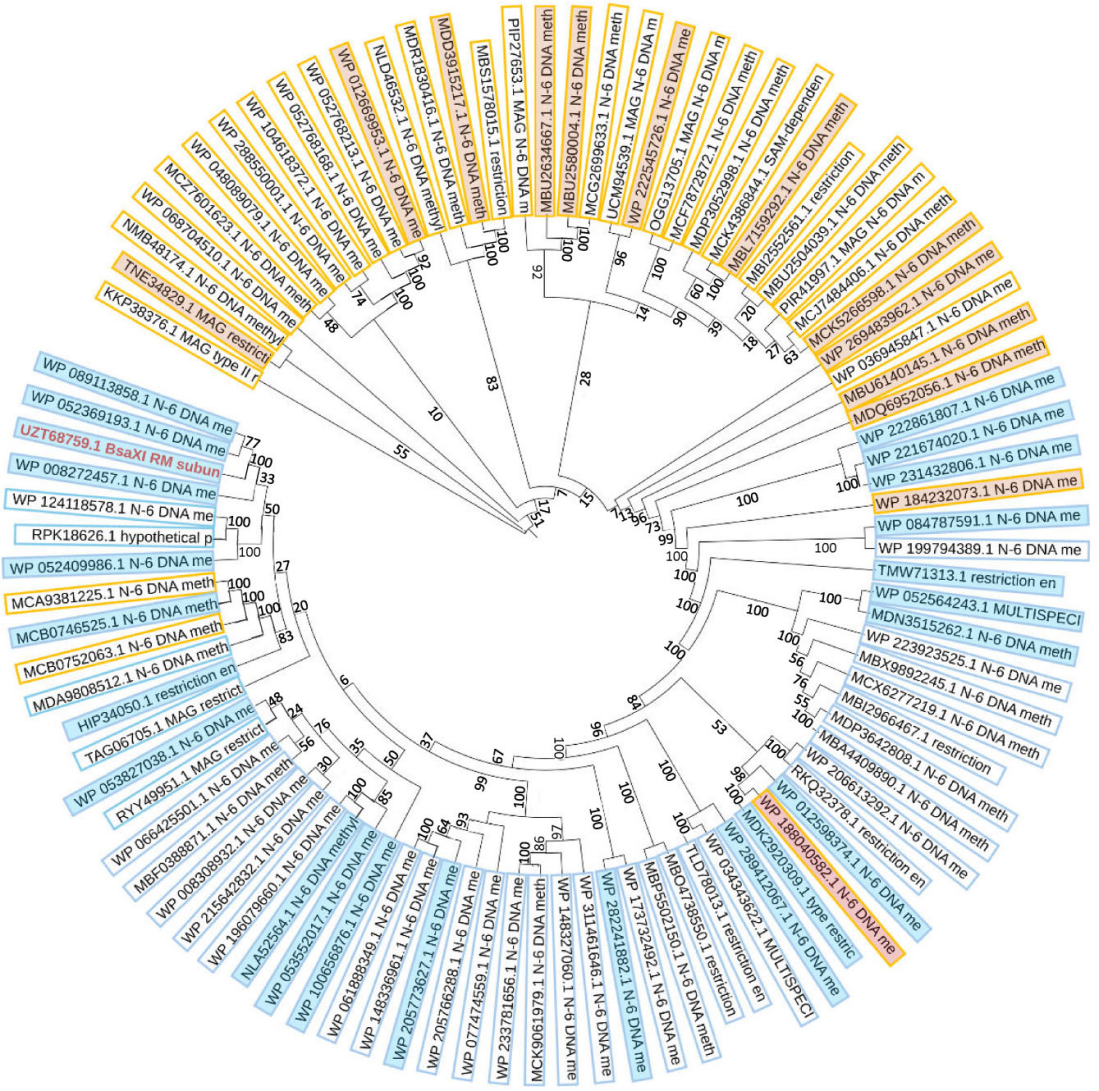
PROMOLS3D based phylogenetic tree of 94 protein sequences that share >50% identity to BsaXI. The orange and blue color wedges indicate protein sequences with < or >800 residues, respectively. The filled orange and blue wedges indicated sequences randomly selected for AlphaFold structure prediction. Results from structure predictions showed that the orange-colored shorter sequences contain, in addition to the highly conserved R, H, and M domains, a knob domain. All shorter entries, with only four exceptions, segregated to a third of the phylogenetic tree circle. The longer protein sequences including BsaXI (in red letters) uniformly contain both the knob and paddle folds, and are phylogenetically further away from the shorter orange species.

In the ‘RMS’ fusion group, all but one (RdeGBIII) contain an archetypical TRD1–CR1–TRD2–CR2 substrate recognition domain at the proteins’ C-terminus (Fig. 8C). The S domain of the RdeGBIII RMS fusion protein (Fig. 8D) resembles that of the Type IIG RMS fusion proteins. BpuSI, and MmeI (Supplementary Table S2).

The AlphaFold predicted BsaXI is the only protein among the previously identified Type IIB enzymes that appears to contain the long antiparallel double helix, that we refer to as a ‘paddle’ (Fig. 8E) and have found (via structural and mutational analyses) to be involved in DNA cleavage. To further examine whether BsaXI represents a novel structural variant of the Type IIB RM subtype, we predicted the structure of 36 putative enzymes from a list of >100 sequences that, based on analyses using the PROMOLS3D webserver [43] displayed better than 50% identity to BsaXI. Results from AlphaFold predictions indicated that all 37, including BsaXI, contain the “knob” element with five short helices wrapped around a long central helix, and more than half (23 out of 36) of the predicted structures contain the paddle domain near the protein C-terminus. The number of turns of the helices on the paddle vary with total number of residues in the sequence, but the tip of the long paddle is invariably rich in D, E, and K residues.

A phylogenetic tree analyses based on PROMOLS3D multiple sequence alignment of 94 putative proteins that bared better than 60% sequence identity with BsaXI, showed that all but 4 BsaXI homologues with <800 residues (Fig. 9; orange outlined or highlighted sequences) segregated to a smaller section of the phylogenetic tree and all sequences containing >850 residues (cyan outlined or highlighted) occupying two third of the phylogenetic circle. All predicted structure of the BsaXI homologues (orange or cyan heighted wedges) contain a multi-helical knob domain but only the longer proteins (cyan heighted wedges) are predicted to contain both the knob and paddle domains. It is reasonable to speculate, based on proven accuracy of AlphaFold predictions and results from the above analyses, that many putative RM enzymes with high sequence homology to BsaXI might likely contain the newly detected knob and paddle domains.

### Stoichiometry of protein assemblage, DNA recognition, and action

2D classification of twice selected particles of both DNA-free and DNA-bound BsaXI particles showed that >95% of selected particles from both sets are single particles with an (RM)_2_S (±DNA) stoichiometry with <5% of the particles in contact with a close neighbor. This observation, together with the fact that adsorption to carbon film in negative-stain electron microscopy ('EM') and flash freezing in cryo-EM preparations capture, rather than alter, conformations of particles, suggests that under the experimental conditions described in this study, BsaXI largely exists in solution, in the presence or absence of bound DNA, largely as a single heterotrimer rather than the higher oligomer state previously reported to represent the predominant solution behavior of the archetype Type IIB enzyme BcgI [13, 14].

However, previous published studies of the cleavage behavior of BsaXI (along with a cohort of additional Type IIB RM systems) have nonetheless indicated that the enzyme is capable of cleaving a plasmid substrate containing two target sites somewhat more rapidly than comparable substrates containing only one target site [15], with the former being cleaved between 1.6- and 4-fold more rapidly under conditions of low or high salt, respectively. This observation has been interpreted as illustrating the enzyme’s need to engage two target sites in order to achieve maximum cleavage efficiency. If true, then such a higher order DNA-bound species {corresponding to [(RM)_2_S-DNA]_2_} is not captured in our negative stain or cryo-EM analyses—possibly due to the fact that such a larger assemblage might only be transiently generated on fully unmethylated sites. In contrast the DNA-bound structure determined here (i.e. in the presence of intrinsically bound SAM and Ca^++^) is incapable of DNA cleavage, possibly due to the methylated DNA duplexes in our enzyme-bound complexes remaining dissociated under our buffer conditions.

Given our results that even hemi-methylation of either strand of the DNA target (as well as methylation of both strands) blocks DNA cleavage on both sides of the bound target, the mechanism of self- versus nonself distinction seems clear. It is likely, similar to other bifunctional RM enzyme systems, that DNA cleavage activity is faster than the methylation, such that invading DNA that is initially unmethylated is therefore cleaved before it is protected. However, any hemi-methylated DNA sites generated in the host during DNA replication would remain resistant to cleavage, and then have a full DNA generation period at their disposal after passage of the DNA replication fork to become fully methylated.

### How does BsaXI generate two precisely defined double-strand breaks?

As described above, BsaXI generates a pair of double-strand breaks at precisely defined distances from the ends of the enzyme’s bipartite recognition site, thereby generating 3 base, 3′ overhangs at both of those positions [17]. Prior biochemical analyses on this enzyme’s mode of action (as well as several additional Type IIB enzymes) have led to general agreement that such enzymes likely bring together at least two bound target sites and then collaborate to complete the process of generating double-strand breaks at either side of each bound target [15]. However, the lack of any visible reaction synapses in our structural studies, along with any obvious route forward to model and generate a realistic higher-order complex containing a “cleavage-ready” complex of REase dimers surrounding the DNA cleavage site, forces us to consider the pros and cons of at least three possible mechanistic scenarios:

Hypothesis 1. Two entirely separate active sites, located at different positions within the RM subunit are individually responsible for individual strand scission events at each cleavage site, thereby producing a double-strand break.

Pros: Several observations, both in the literature for other phage restriction systems and in our own analyses reported here, would appear to support such a hypothesis. Several DNA cleaving enzymes (including CRISPR-Cas9 [44], the Type IIS restriction endonuclease BspQI [45], single-chain Type IIT endonucleases such as Bpu10I [46], and single chain LAGLIDADG homing endonucleases such as I-SceI [47] and I-AniI [48]) contain two separate nuclease active sites, that can be mutationally uncoupled from one another to create corresponding nicking enzymes. In the structures of BsaXI presented here, two quite separate structural domains and motifs are located near each cleavage site (the N-terminal REase domain and the C-terminal double-helical “paddle”). Within both of those regions, individual point mutations can lead to inactivation of the enzyme, implicating each structural motif in DNA hydrolysis.

Cons: In our experiments, none of the point mutations placed individually into the two structural domains or motifs noted above provide an unambiguous demonstration that inactivation of a single active site leads to a site-specific nickase, as is the case for all the previously described systems noted above. In addition, the specific residues in the paddle that might be involved in catalysis (E, D, and K residues between residues 810 and 825, and most specifically D816 and E823) are not obviously conserved across a group of 96 recognizable Type IIB homologues, beyond the two closest homologues [WP_052369193.1 (N-6 DNA methylase from *Geobacillus kaustophilus*), and WP_089113858.1 (N-6 DNA methylase from *Geobacillus thermocatenulatus*)]. While it is formally possible that BsaXI and these two most closely related enzyme represent an unappreciated, mechanistically and structurally unique subtype of the group IIB enzymes, it would seem more likely that the ‘paddle’ region of BsaXI and its most immediate cousins (which forms a helical bundle motif generally associated with protein–protein association, rather than a nuclease active site) represents a novel structural elaboration that plays an indirect role in DNA cleavage, perhaps by facilitating transient interactions between endonuclease domains and DNA of individual bound complexes.

Hypothesis 2. The N-terminal endonuclease domain is solely responsible for cleavage of both strands at each cleavage site, without the need for the formation of a higher order enzyme-DNA complex, via a mechanism where it independently and sequentially attacks each scissile phosphates within a single cleavage site, by virtue of a long linker that allows for it to extend to each site of hydrolysis.

Pros: There is no doubt that the REase domain is in fact involved in DNA hydrolysis; a cluster of four catalytic residues in that domain identified via mutagenesis and cleavage assays (E73, D90, E105, and K107) are absolutely conserved across the entire larger family of Type IIB described above. In addition, there is at least one precedent for significant motion of an endonuclease domain during DNA cleavage by a restriction endonuclease (PaqCI) via an extended linker, to cleave at significant distance to one side of that enzyme’s bound target, either in *cis-* or in *trans-* [33, 34].

Cons: It is very difficult to envision how, if a single flexible REase domain is cutting both DNA strands to generate a double-strand break, how it could do so in a manner that generates a precise 3 base, 3′ overhang rather than nicking each DNA strand at multiple sites that are limited only by the extent of reach of that domain’s linker. As well, in published studies using BsaXI as well as multiple additional Type IIB RM systems, none of those enzymes displayed significant accumulation of an early relaxed circle (open circle) intermediate that would be indicative of individual, sequential strand scission events—even when using plasmids substrates containing two target sites, corresponding to eight sites of DNA strand scission over the course of the reaction digest [15].

Hypothesis 3. Two endonuclease domains are brought together in a transient catalytic dimer at each site of DNA cleavage.

Pros: As mentioned above, this mechanistic scenario enjoys the advantage of having been previously tested biochemically for BsaXI and its closest known RM systems—experiments that led to the general conclusion that cleavage likely involved concerted interactions between two DNA-bound enzyme complexes [15, 16]. This possibly also has additional precedent, specifically for many Type IIS/IIG enzymes visualized to-date on DNA (such as BfiI [49], FokI [50], PaqCI [33], and DrdV [34]) that cleave at a distance from the bound target. Beyond those previously described studies, a requirement for REase dimerization for cleavage to occur would allow us to more readily explain how the enzyme generates well-defined, precisely positioned 3-base, 3′ overhangs at each cleavage site: the cleavage pattern would be dictated by the relative packing of the two REase domains in their dimeric organization and packing against the DNA cleavage site, as was previously described for the Type IIS systems noted above. As well, this mechanism might explain why some mutations in the paddle domain inhibit cleavage: it is possible that region, which is obviously located near the DNA cleavage site, might be a participant in the formation of a transient catalytic dimer at the site of cleavage.

Cons: As mentioned above, we have found no evidence in either the solution behavior of the protein prior to our structural studies, or via the presence of a population of single particles corresponding to higher-order protein–DNA assemblages on EM grids, in either the presence of absence of DNA. If such higher order structures are in fact generated during cleavage, we have failed to capture them, due to either the use of a buffer composition that is incompatible with the formation of a higher order DNA-bound assemblage, or the presence of a DNA methylation state that is also incompatible with such a higher order bound state (and to reiterate, the endonuclease domains in our structures, while being located near the cleavage sites, are not bound in an intimate complex with the scissile phosphate in our structures).

Beyond the lack of such structural indications of a higher-order assemblage that brings together multiple DNA-bound enzyme complexes, it has also proved difficult to satisfactorily model two DNA-bound BsaXI complexes coming together to form a higher order structure that puts two REase domains together over a cleavage site. In contrast, in prior structural analysis of the Type IIS(G) RM enzyme DrdV, cryo-EM structures very clearly indicated the generation of higher order DNA-bound enzyme complexes that produced REase dimers at the site of cleavage. As noted earlier, the AlphaFold predicted structures of the S-subunit of three identified Type IIB enzymes, including *S.BcgI, S.BveI, S1.HpyUM and S2.HpyUM*, displayed either incomplete or scrambled TRD1–CR1–TRD2–CR2 configurations. It is possible that the lack of CR2 and/or crisscrossing of the CR1/CR2 double helices prevented the formation of the four-helix bundle with the TTC helix of the RM subunits in the formation of single (RM)_2_S particles and necessitated the higher order oligomerization of these enzymes upon activation.

## Supplementary Material

gkaf291_Supplemental_Files

## Data Availability

The coordinates, cryo-EM maps and validation reports for the structures reported in this study are available for immediate download by the public. PDB (https://www.rcsb.org/): DNA-free structure = 8W0P; the two DNA-bound conformers = 8W2P and 8W2Q. EMDB (https://www.ebi.ac.uk/emdb/): DNA-free structure = EMD-43711, the two DNA-bound conformers = EMD-43754 and EMD-43755.
