# Supplementary material for: Cryo-EM structures of DNA-free and DNA-bound BsaXI: architecture of a Type IIB restriction–modification enzyme
Source: Nucleic Acids Res. 2025 Apr 15;53(7):gkaf291. doi: 10.1093/nar/gkaf291 (PMC11997821; doi:10.1093/nar/gkaf291)
Supplement: gkaf291_Supplemental_Files [file gkaf291_supplemental_files.zip › Supplementary_Materials_Text.pdf]

Supplementary Materials

Supplementary Movie Legends

Supplementary Movie 1. Top (Panel a): 3D variability components of the ”Open” conformation of the DNA free BsaXI map viewed down an axis perpendicular to the CR1-CR2 double helix, showing the twisting of the S subunit and “breathing” of the RM subunits. Bottom (Panel b): Same 3DVA components as above except the map was turned 90° toward the top of the page, showing the up-and-down bending of the S subunit and the extension/contraction of the paddle double helices.

Suppelmentary Movie 2. Side-by-side comparison of the CryoEM structure of the “Open” DNA free (left) and the “Closed” cognate DNA bound (right) BsaXI particles.

Supplementary Movies 3a and 3b. Speculation on the transition of a hypothetical ideal B-form DNA docked “open” BsaXI to the cryoEM characterized ”closed” BsaXI/DNA complex conformations. ***Movie a***: AC site of an ideal DNA target docked close to the TRD1 of the apoBsaXI. ***Movie b:*** CTCC site of an ideal DNA target docked close to TRD2 of the apoBsaXI.

Supplementary Movies 4a and 4b. 3D Variability/Display of the two conformations of the BsaXI-DNA particles. The maps were aligned so that the S subunits and the DNA double strands were in the same orientation. The TRD1 and the 5’-AC site are at the top whereas TRD2 and the CTCC are at the bottom. It is the REase close to the 5’-AC site that The movies showed clearly the difference in the density level corresponding to the REase in the two conformers, and that it is the REase domain close to the 5’-AC site that varied in the 3D landscape of the particles. Furthermore, both REase on the top of both conformers were in close contact with the DNA at times.

Supplementary Figure Captions

Supplementary Figure S1. Preparation and preliminary analyses of BsaXI electron micrographs. *Panel a:* SuperDex200 SEC chromatography profile of Heparin purified BsaXI RM/S complex (A, left panel). Fraction #12 was concentrated, flash froze in small aliquots and stored at -80°C; Overlay of BioRad Enrico650 SEC chromatography profiles of DNA free (red) and cognate DNA bound BsaXI complex (blue) (A, Right panel). Blue dots are ratio of OD260/OD280; Insets, ns-TEM image of DNA free (A, left) and cognate DNA bound BsaXI particles (A, right). *Panel b:* Preliminary assessment of project feasibility: Left panels, images of negative stained specimens of BsaXI from FN20 with (top) and without (bottom) manual picking circles. Middle panel: 2D classification of manual picked particles (top). Red outlined images are selected classes for template picking and 2D classification of 16370 template-picked/curated

particles (bottom). Red outlined classes were particles selected for *ab-initio* one model reconstruction. Right panels: Two different views of the ~15Å resolution molecular envelop reconstructed from 12,800 selected particles from ns-TEM movies. C. Preliminary assessment of project feasibility: Six initial screening datasets with a total of 2508 movies were collected at 1.16 Å pixel size using a FEI GLACIOS electron Microscope. The particles were picked using the selected negative stained images as template. After two rounds of 2D classification and 2 D selection, 294,772 particles were used for 3D reconstruction of four model. Classes *i* and *iv* were refined by homogenous, non-uniform, and local refinements. D Three different views of the 4.5 Å resolution map reconstructed/refined from Six initial screening datasets with a total of 2508 movies collected at 1.16 Å pixel size using a FEI GLACIOS electron Microscope. After preprocessing (C-I) particles were picked using the selected negative stained images as template. After two rounds of 2D classification and 2 D selection (C-II), 294772 particles were used for 3D *ab initio* reconstruction for four models (C-III). Classes C-III-*i* and *iv* were refined separately to 4,52 Å and 5.86 Å, respectively corresponding to the $(RM)_2S$ complex (*i)* and the RM monomer (*ii*) (C-IV). Different views of the refined (C-V) and segmented $(RM)_2S$ map (C-V and C-VI, respectively).

Supplementary Figure S2. Flow chart of DNA free BsaXI data processing. 6227 movies were collected at a super resolution of 0.5395 A/pixel using KRIOS #4 at PNCC. After preprocessing, 500 out of the accepted 5965 movies were use for blob particle picking with the maximum and minimum diameter of 200 and 80 respectively and a minimum separation of 1 diameter. After inspection, 301,422 particles were extracted and subjected to two rounds of 2D classification/Selection. 11 out of 100 classes were selected and used as templates for template picker (diameter 200 Å). Following inspection, extraction, and two rounds of 2D/Classification and 2D selection, 1,061,526 particles were used for a four-model *ab-initio* 3D reconstruction (I). Only class iv of the reconstructed models bearded resemblance to the initial 4.5 Å model generated from the screening dataset by *ab initio* 3D reconstruction and refinement of a single model (Figure S1,Panel C) and was used for refinements. Which yielded a map with 3.38Å at a GSFSC of 0.143 (II). The refined model with 362,285 particles was subjected to two rounds of heterogenous refinements (III and IV) to further eliminate bad particles resulting in two classes with similar images except distribution of sporadic densities near the edges. In the final round of local refinement, a mask was introduced to eliminate the signals contributed to the sporadic density and resulted in a final model at 3.26 Å resolution (v).

Supplementary Figure S3: Flow chart of DNA bound BsaXI data processing and refinements: Data collected on an in-house Glacios electron microscope at a super resolution of 0.56 Å/pixel (A) and on a PNCC Krios electron microscope at a slightly higher resolution of 0.528 Å/pixel (B) were processed, reconstructed and refined separately to 3.11 Å and 3.22 Å respectively. The particles used for refinement from the two datasets with slightly different pixel sizes were re-extracted and combined using parameters provided by the script Boxscaler.py (A-V and B-V).

The combined particles stacks were used to refined volume (*ii)* from B-IV and resulted in volume *iii* with a resolution of 3.05 Å (C-I, left). However, there were weak densities of helix at both ends of the antiparallel double helix (red arrows. C-I, left) and 3D variability analysis/Display showed that there were two different populations of particles in the 3D landscape (C-I right panels). Heterogenous refinement showed that there were small amounts of unbound subunits or fragments (*iv, v*) and two major classes with different configurations (volumes *vi and vii*). Refinements of volumes *vi* and *vii* separately resulted in two different conformations of the DNA bound BsaXI complex (volumes *viii and ix*). The red circles and rectangle outlines in C-IIIa and C-IIIb showed clear density for a helix at the end of CR1 but none at CR2, which is consistent with the DNA free BsaXI $(RM)_2S$ map. 3D variability analysis/Display of volume *ix* showed that there were only one population of particles in the 3D landscape (C-IV). Same results were obtained for volume *viii* (not shown).

Supplementary Figure S4. ***Panels a and b:*** Amino acid sequence and visualized secondary structures of the RM (Panel a) and S (Panel b) subunits. The green and blue lighting bolts indicate the interruption and continuation of secondary structure elements. Residues with magenta underline in panel a represent the secondary structural elements in the highly conserved MTase fold while residues between the orange brackets belong to the Knob domain.

Supplementary Figure S5: Structural comparison of N6-adenine-methyltransferase containing RM nucleases. Ribbon model of RM.BsaXI (A), DrdV (B), MmeI (C), BpuSI (D), M.TaqI (E) and M.EcoKI124 (F) were aligned based on the N6-Adenine-methylase and displayed alongside with the Nuclease (REase), Helical Connecter (HC) and Target recognition (TRD) domains when available. (G) Schematic representation of the MTase domain. It clearly showed that despite the differences in spatial distribution and fold of other domains, the structure of the N6-Adenine-methyl transferase is highly conserved with five helices (colored blue) franking the 8-strand central beta-sheet (green), three in the front (Solid outline) and two in the back (dashed outline) viewed according to the display of the ribbon models. Seven of the beta-strands are parallel with strand #7 inserted between strands #5 and #6 and running antiparallel to the others. The stretch between the C-terminal of strand #7 and the N-terminal of beta-strand #8 is the place for insertion of sub-domains, large or small. In the MS or RMS fusion of Type II R/M systems, the TRD domain is usually extended from a ninth beta-strand attached antiparallel to the 8th strand of the central beta-sheet (B,C,D,E). In BsaXI, the N-terminal beta-strand of the TRD domains in the S-subunit is attached antiparallelly to strand #8 of the MTase of the RM subunits.

Supplementary Figure S6. ***Panel a:*** Local resolution distribution of the initial BsaXI-DNA complex map at three different orientations, showing the low*.* ***Panel b:*** Specific contacts between amino acid residues (colored in grey) in the S-subunit and bases (beige and orange) at and/or near the boxed target sites. The colored bases in the sequence shown on top are the ones featured in the panels. Hydrogen bonds

between the base pairs are not shown. ***Panel b i:*** Y169 insert into the space emptied by the flipped-out adenine and hydrogen-bonded to the upstream adenine (beige) and the left behind lone thymine (orange) at the upstream AC site. ***Panel b ii:*** R112 forming 4 H-bonds to the two Guanine bases compliment to the cytosines immediately downstream of the flipped-out adenine. ***Panel b iii:*** The beta hydroxyl and carbonyl oxygens of T416 forming three HBs to the first C:G base-pair of the downstream CTCC target; ***Panel b iv:*** K419 of the S-subunit and K761 of the RM subunits, (either A or B subunit, depends on whether in conformer I or II) stabilizing the base pair immediately after the flipped-out base in the downstream CTCC site. ***Panel b v:*** R351 forming two HB to the complement guanine of the last base-pair in the CTCC half site.

Supplementary Figure S7. ***Panel a:*** Overlay of ribbon models of a representative RM subunit from the DNA free (colored grey) and the two individual RM subunits (green and purple) from conformer I. The RM* model in green showed a gradually increasing conformation change propagated from the center of the subunit toward the N-terminal half of the molecule which is trigged by interactions with the adenine base in the active site of its MTase domain. Whereas the conformation of the other RM subunit (purple) remained relatively unchanged from the DNA free molecule (grey). It also showed that a slight change in the direction of the $P_{363}TP_{365}$ platform led to a slight tilt in the strand connecting the MHC and the first helix of the MTase domains. Which in turn resulted in a downward pull of the mHC and led to a big shift in the position of the REase domain. Inset shows the tilting of the beta strands at residue P363 (red arrowhead). ***Panels b & c:*** Densities around the flipped-out unmethylated adenine (***b***, left) and the 6-methyl-adenine (***b***, right); and densities around the cofactors SAM (***c***, left) and SAH (***c***, right). The arrows in ***panel c*** indicated the presence and absence of methyl group at the SD atoms of the cofactors SAM and SAH. ***Panel d:*** Superposition of an ideal B-form DNA (B-DNA) target sequence (colored yellow and orange) with the DNA target (colored cyan and purple) of a BsaXI-DNA complex. For clarity, the S subunit in front of the model is not shown; ***Panel e:*** Superposition of the model of an ideal B-form DNA and the cryoEM coordinates of the DNA target in a BsaXI-DNA complex, showing the excellent overlay of the variable base pairs between the two flipped out bases (left) and the shortening of the distance between the phosphate atoms from 34.3 Å in the B-DNA to 30.4Å for the DNA target in the complex (right).

Supplementary Figure S8. Panels a and b. Two hypothetical BsaXI-B-DNA "initial binding complex" models from SSM superposition of RM subunit A or B with the RM* subunit in either BsaXI-DNA conformer I (***Panel a***) or conformer II (***Panel b***), respectively. In ***Panel a***, the 5'-AC site is closer to TRD1, while in ***Panel b***, the downstream CTCC (or its GAGG complement) is closer to TRD2. See Movie 4 for the morphing between a speculative "open" hypothetical "initial binding complex" and the cryoEM structure of the two conformers of the "Closed" BsaXI/DNA complexes. ***Panels c & d:*** Structural Comparison of Type I and Type IIB restriction modification system in complex with DNA. *Panel **b***.

CryoEM structure of the Type IIB R/M enzyme BsaXI in complex with a 34 bp oligo duplex containing the specific bipartite target site; ***Panel d***, CryoEM structure of the archetype Type I EcoKI124 in complex with a 36 bp non-canonical double strand oligo. Shown in tan, blue and green are the two separate RM and S subunits, respectively. The grey ribbons are the missing REase density in one of the RM subunit (***c***) or the docked R subunit of the Type I EcoKI124 (***d***).

Supplementary Figure S1

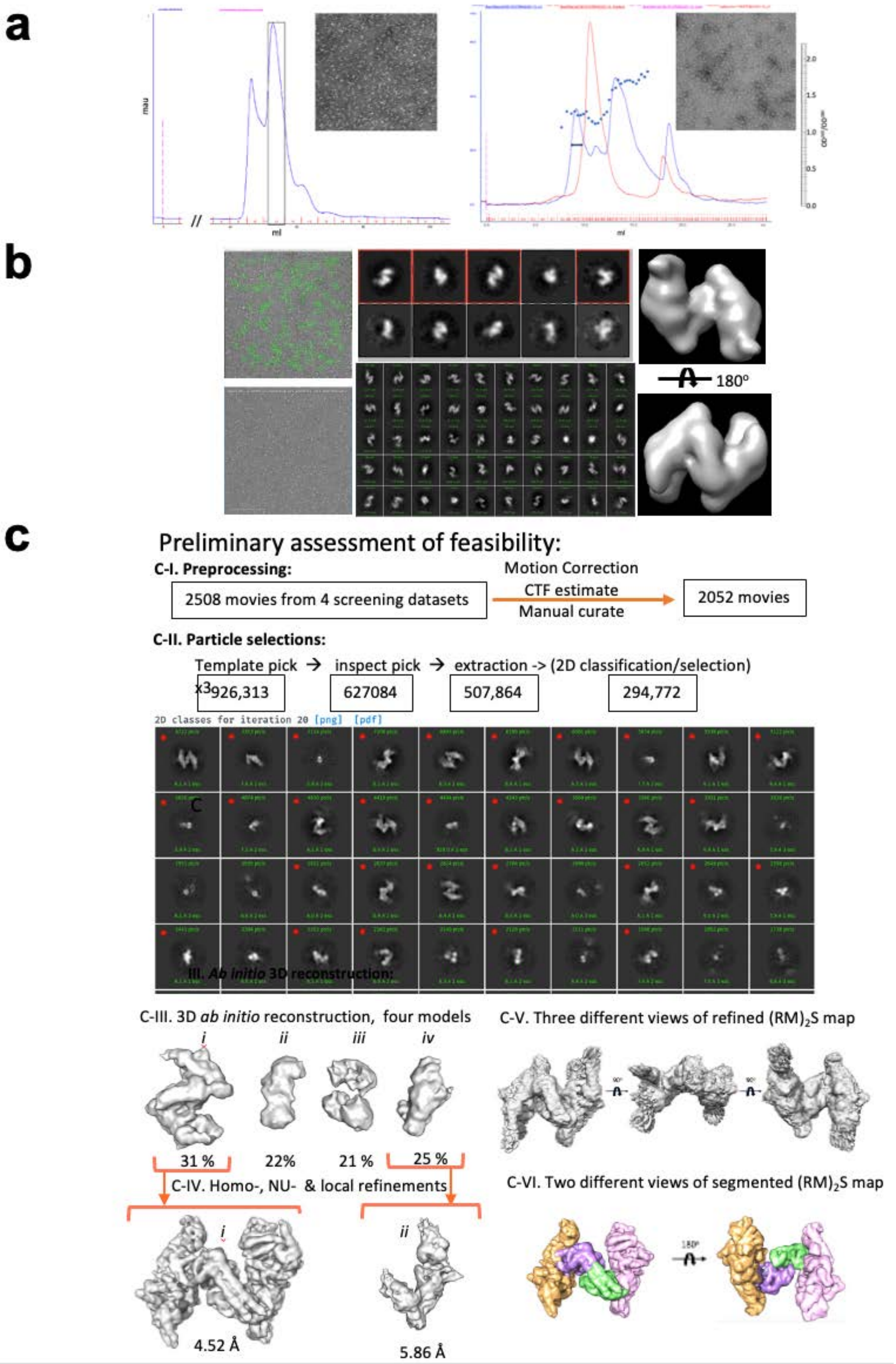

Supplementary Figure S2

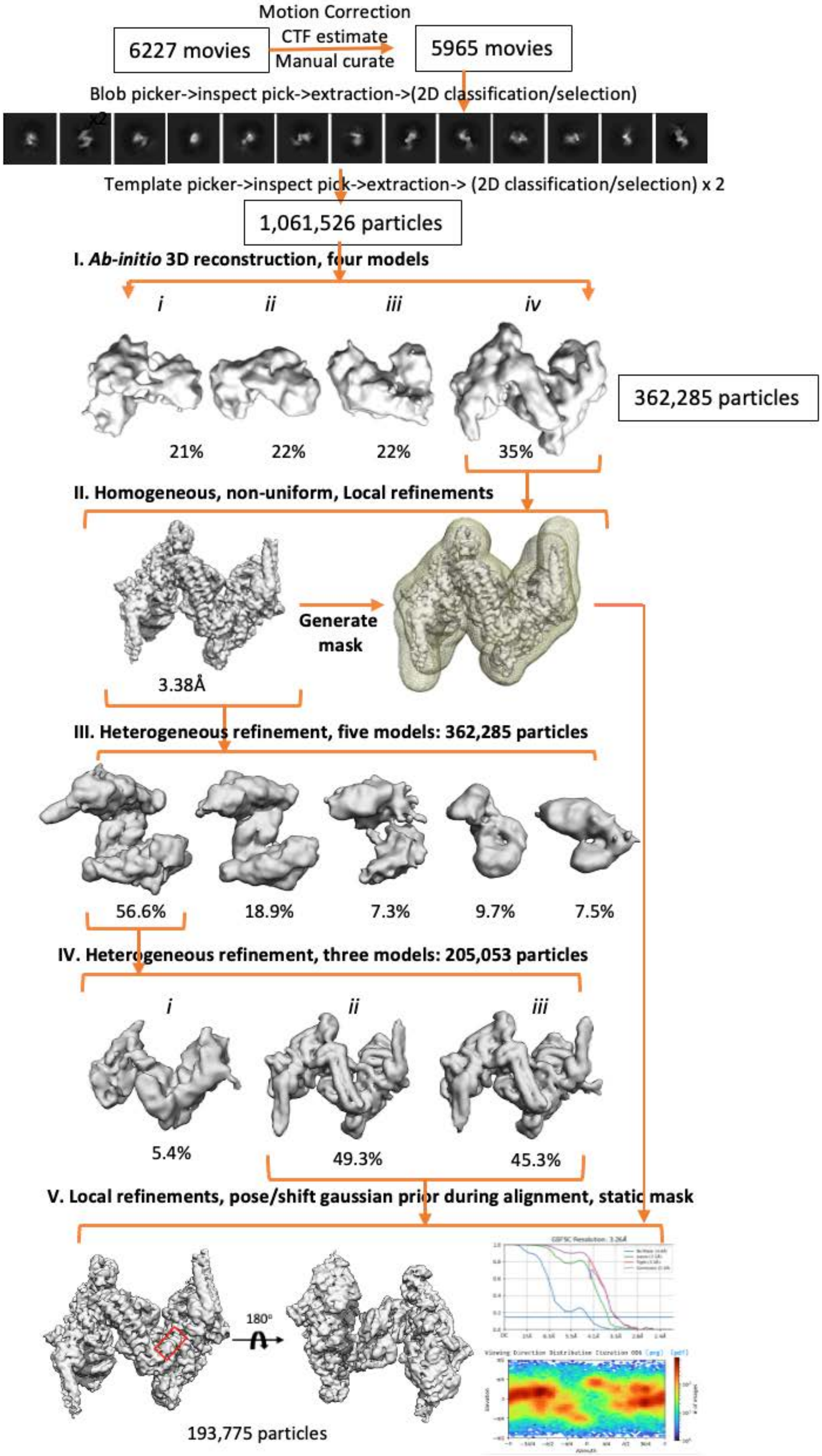

Supplementary Figure S3

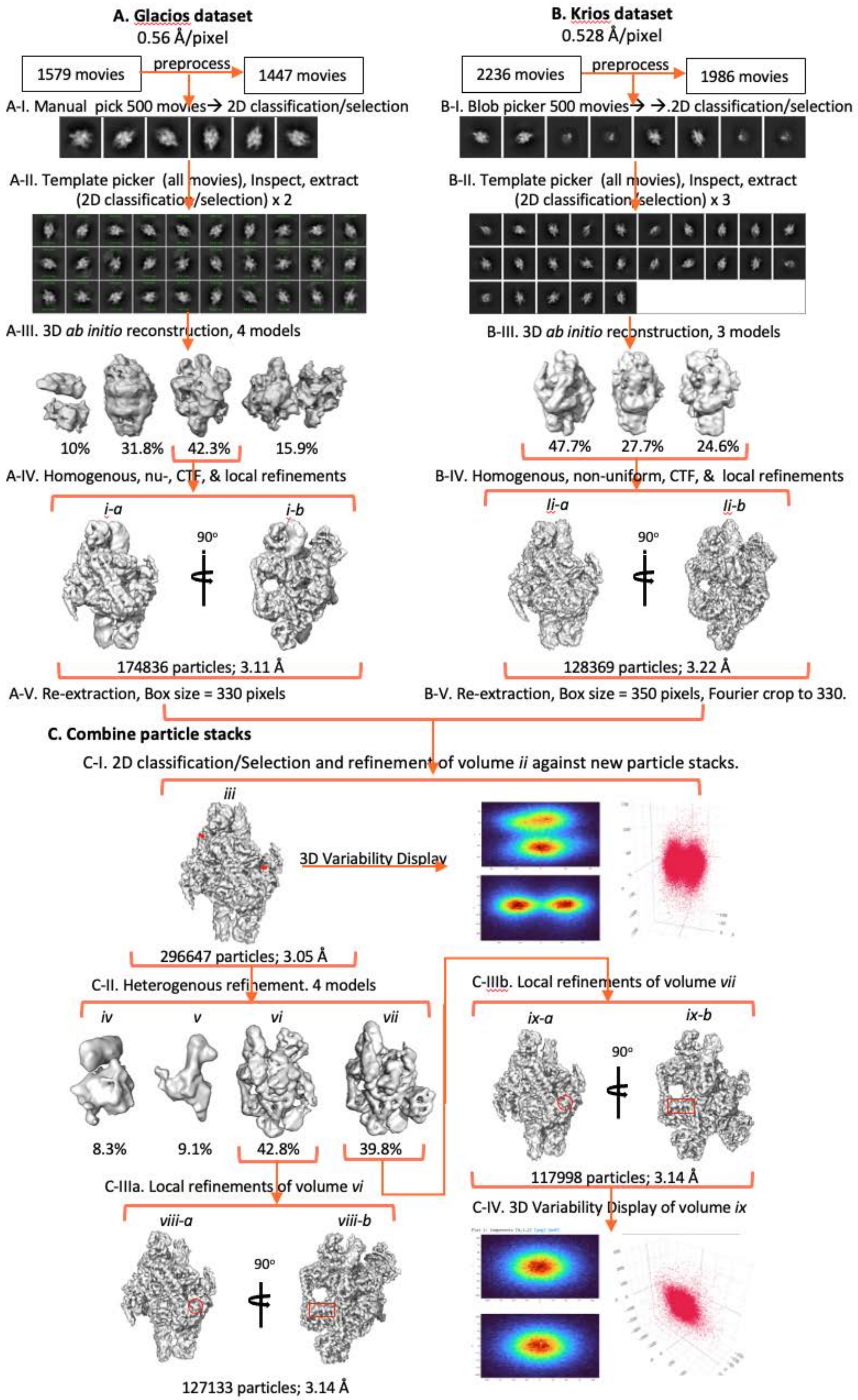

Supplementary Figure S4

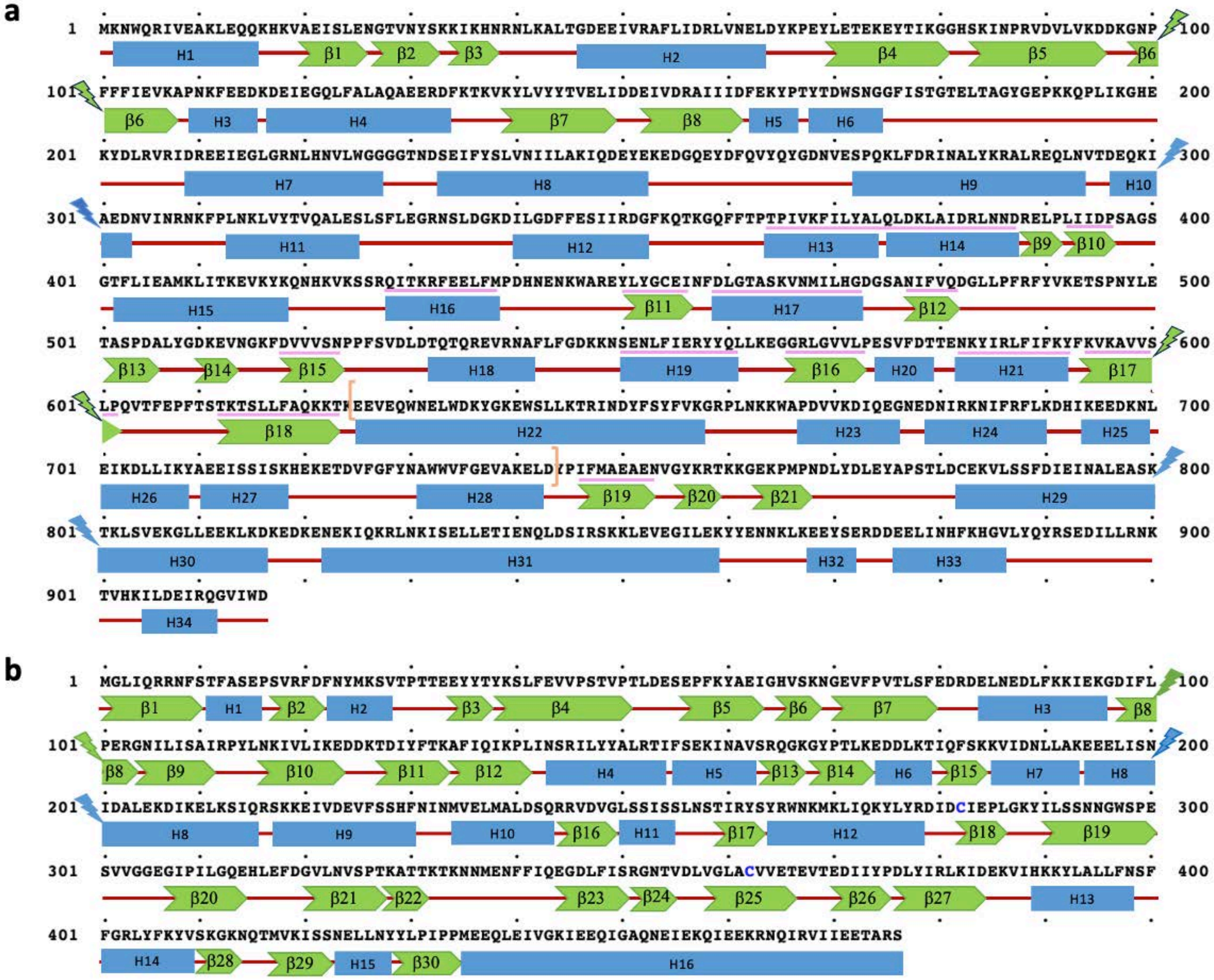

Supplementary Figure S5

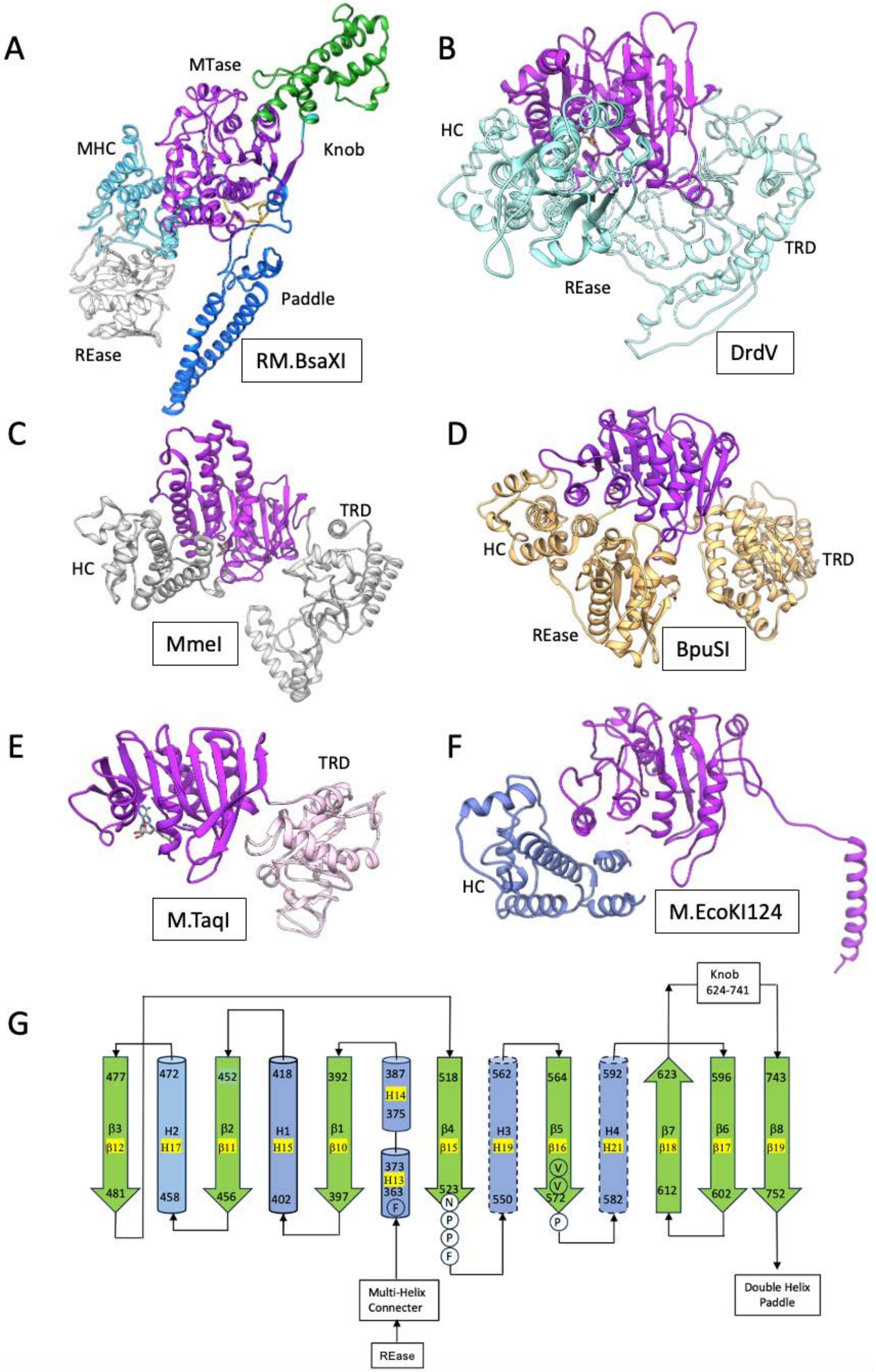

Supplementary Figure S6

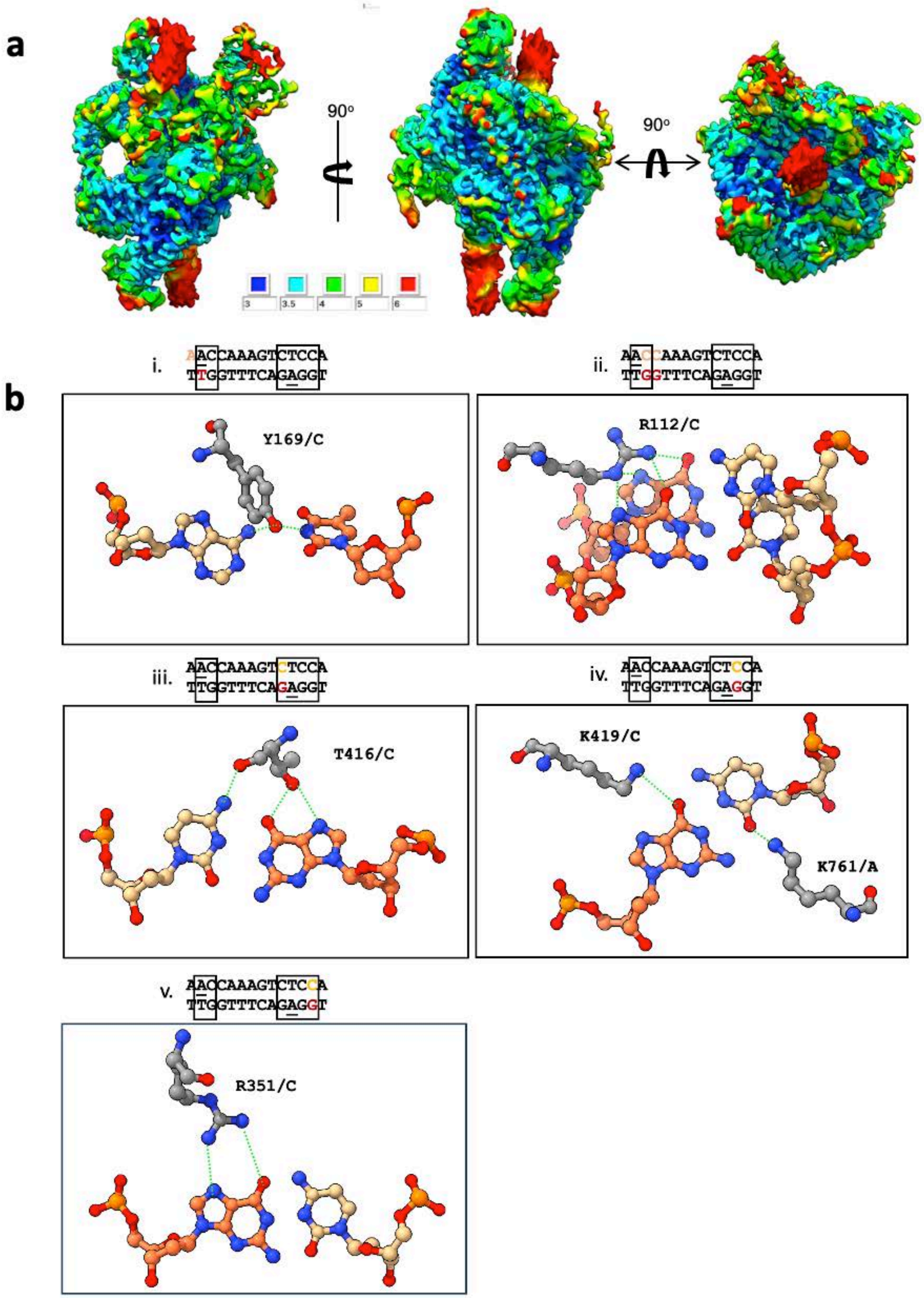

Supplementary Figure S7

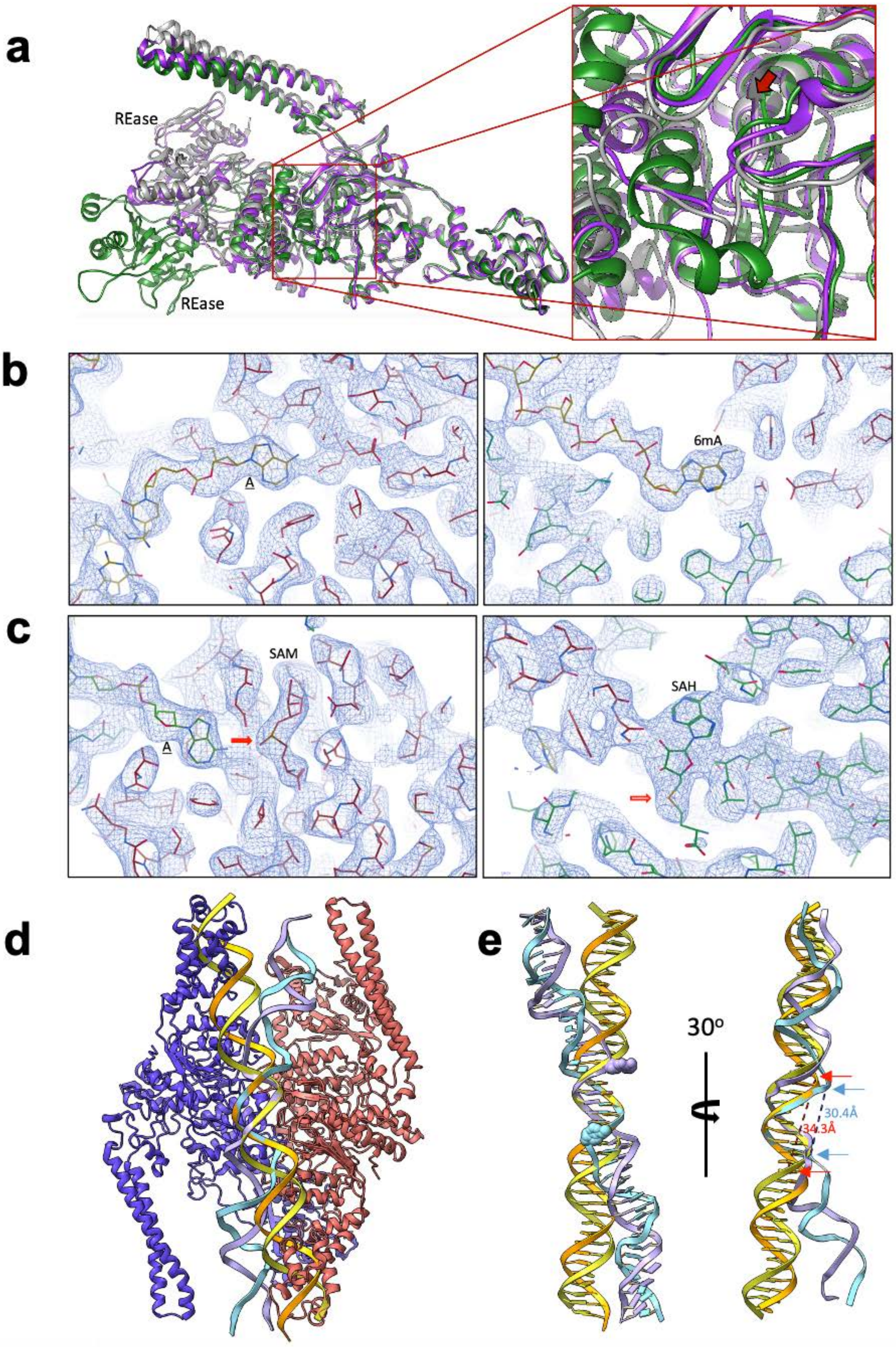

Supplementary Figure S8

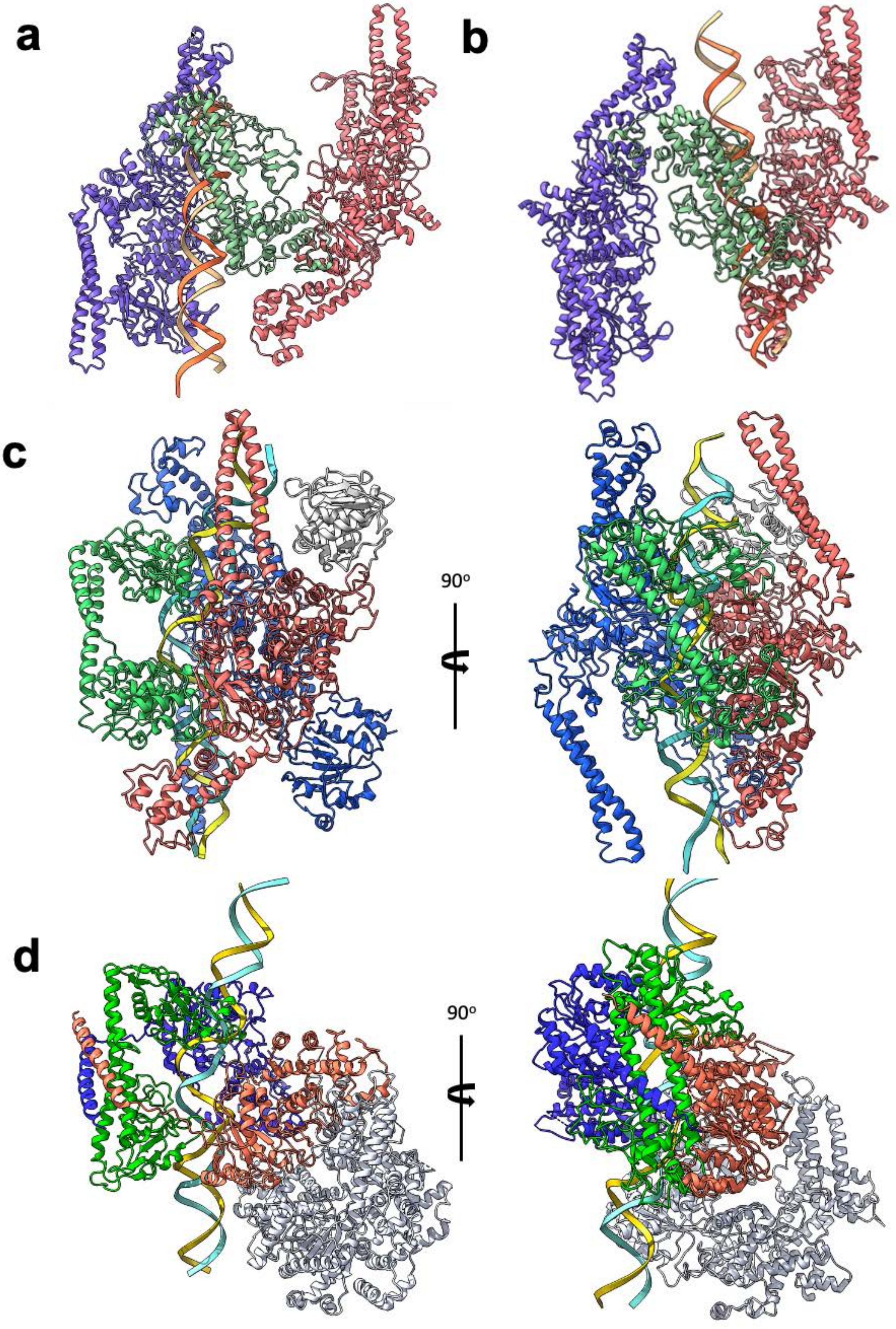

Supplementary Table S1. Mutagenesis Oligos

BsaXI RM subunit N-terminal mutants

D57Af	GCGCGATTGGTAAATGAACTG
D57Ar	TATTAAGAATGCTCTTACTATCTCTTCATCACCA
D64Af	GCGTACAAACCTGAGTATTTGGAAAC
D64Ar	CAGTTCATTTACCAATCGGTCTATTAAGAA
E71Af	GCGACCGAGAAGGAGTACACTATTAA
E71Ar	CAAATACTCAGGTTTGTAATCCAGTTCATT
E73Af	GCGAAGGAGTACACTATTAAGGGCG
E73Ar	GGTTTCCAAATACTCAGGTTTGTAATCC
E75Af	GCGTACACTATTAAGGGCGGTCA
E75Ar	CTTCTCGGTTTCCAAATACTCAGGT

BsaXI RM subunit C-terminal mutants

E811Af	GCGGAGAAATTAAAGGATAAAGAAGATAAAG
E811Ar	CAAGAGCCCCTTCTCAACAGATAA
K813Af	GCGTTAAAGGATAAAGAAGATAAAGAAAATGA
K813Ar	CTCTTCCAAGAGCCCCTTCTCA
D816Af	GCGAAAGAAGATAAAGAAAATGAAAAAATACA
D816Ar	CTTTAATTTCTCTTCCAAGAGCCCC
E818Af	GCGGATAAAGAAAATGAAAAAATACAAAA
E818Ar	TTTATCCTTTAATTTCTCTTCCAAGAGCC
K820Af	GCGGAAAATGAAAAAATACAAAAGAGA
K820Ar	ATCTTCTTTATCCTTTAATTTCTCTTCCAAGAG
E821Af	GCGAATGAAAAAATACAAAAGAGACTCA
E821Ar	TTTATCTTCTTTATCCTTTAATTTCTCTTCCAAGA
E823Af	GCGAAAATACAAAAGAGACTCAATAAAATATCTG
E823Ar	ATTTTCTTTATCTTCTTTATCCTTTAATTTCTCTTCC

Second round of mutagenesis to varify putative PD/ExK motifs

D90Af	GCGGTATTAGTAAAGGATGATAAGGGG
D90Ar	CACTCTTGGATTTATCTTACTATGACCGC
D96Af	GCGAAGGGGAATCCATTCTTT
D96Ar	ATCCTTTACTAATACATCCACTCTTGGATTTATC
E105Af	GCGGTAAAAGCTCCAAACAAATTC
E105Ar	AATAAAAAAGAATGGATTCCCCTTATCATC
K107Af	GCGGCTCCAAACAAATTCGAG
K107Ar	TACTTCAATAAAAAAGAATGGATTCCCC
E114Af	GCGGACAAAGATGAGATTGAAGG
E114Ar	CTCGAATTTGTTTGGAGCTTTTACTTC
K116Af	GCGGATGAGATTGAAGGTCAG
K116Ar	GTCTTCCTCGAATTTGTTTGGAGC

Supplementary Table S2. Type IIB AlphaFold structural predictions

Enzyme[a]	Recognition sequence	# residues	Predicted Fold	Predicted C-ter fold	pTM
AjuI	(7/12) GAANNNNNNNTTGG (11/6)	NA			
AlfI	(10/12) GCANNNNNNTGC (12/10)	NA			
AloI	(7/12) GAACNNNNNNTCC (12/7)	1262	RMS	TRD1-CR1-TRD2-CR2	0.695
ArsI	(8/13) GACNNNNNNTTYG (11/6)	NA			
BaeI.RM	(10/15) ACNNNNGTAYC (12/7)	626	RM, Type I-like	CTT	0.88
BarI	(7/12) GAAGNNNNNNTAC (12/7)	NA			
BceIV.RM	(7/5) GCAGC (9/11)	382	RM, Type I-like	CTT	0.888
BcgI.RM	(10/12) CGANNNNNNTGC (12/10)	637	RM, Type I-like	CTT	0.886
BdaI	(10/12) TGANNNNNNTCA (12/10)	NA			
BplI	8/13) GAGNNNNNCTC (13/8)	NA			
BsaXI.RM	(9/12) ACNNNNNCTCC (10/7)	916	RM/S[c]	Double-Helix Paddle	0.864
Bsp24I	(8/13) GACNNNNNNTGG (12/7)	NA			
Bve1B23I.RM	GACNNNNNTGG	614	RM, Type I-like	CTT	0.792
CjeI	(8/14) CCANNNNNNGT (15/9)	1273[b]	RMS	TRD1-CR1-TRD2-CR2	0.703
CjePI	(7/13) CCANNNNNNNTC (14/8)	1296[b]	RMS	TRD1-CR1-TRD2-CR2	0.721
CspCI	(11/13) CAANNNNNGTGG (12/10)	NA			
FalI	(8/13) AAGNNNNNCTT (13/8)	NA			
HaeIV	(7/13) GAYNNNNNRTC (14/9)	953	RMS	TRD1	0.806
Hin4I	(8/13) GAYNNNNNVTC (13/8)	NA			
HsoII	(8/14) CAYNNNNNRTG (14/8)	1303	RMS	TBD1-CR1-TBD2-CR2	0.836
NgoAVIII	(12/14) GACNNNNNTGA (13/11)	274	MTase?	?	0.78
NmeDI.RM	(12/7) RCCGGY (7/12)	351	RM?	?	0.826
PcoI*	( ) GAACNNNNNNTCC ( )	1297	RMS	TRD1-CR1-TRD2-CR2	0.67
PpiI	(7/12) GAACNNNNNCTC (13/8)	1289	RMS	TRD1-CR1-TRD2-CR2	0.747
PsrI	(7/12) GAACNNNNNNTAC (12/7)	NA			
RdeGBIII	(9/11) TGRYCA (11/9)	1109	RMS, BpuSI-like	Beta Barrel	0.864
SdeOSI.RM	(11/13) GACNNNNRTGA (12/10)	630	RM, Type I-like	CTT	0.876
TstI	(8/13) CACNNNNNNTCC (12/7)	1251	RMS	TRD1-CR1-TRD2-CR2	0.687
UcoMSI	(7/5) GAGCTC (5/7)	NA			
HpyUM032XIII.RM*	( ) CYANNNNNNTRG ( )	679	RM, Type I-like	CTT	0.84
Hso63250II.RM*	( ) CGANNNNNRTAY ( )	680	RM, Type I-like	CTT	0.836
BmuSORF1564P*	( ) GAGNNNNNGT ( )	1134	RMS	TRD1-CR1-TRD2-CR2	0.728

[a] All entries from REBASE website Type IIB except as indicated. NA = Not Available.
[b] Sequence provided by Dr. Gorge M. B. Vitor.
[c] CryoEM structure from this work.
* Enzymes not listed as Type IIB in REBASE but recognize bipartite substrates.
Bases (or complements) in red denote known 6-methyl-adenine methylase sites
